# Synthesis and Applications of Porphyrin-Biomacromolecule Conjugates

**DOI:** 10.3389/fchem.2021.764137

**Published:** 2021-11-08

**Authors:** Pravin Pathak, Mohammad Amin Zarandi, Xiao Zhou, Janarthanan Jayawickramarajah

**Affiliations:** ^1^ Department of Chemistry, Tulane University, New Orleans, LA, United States; ^2^ Department of Biochemistry and Molecular Biology, Tulane University, New Orleans, LA, United States

**Keywords:** bioconjugation, porphyrin bioconjugates, DNA-porphyrin, peptide-porphyrin, protein-porphyrin, antibody-porphyrin

## Abstract

With potential applications in materials and especially in light-responsive biomedicine that targets cancer tissue selectively, much research has focused on developing covalent conjugation techniques to tether porphyrinoid units to various biomacromolecules. This review details the key synthetic approaches that have been employed in the recent decades to conjugate porphyrinoids with oligonucleotides and peptides/proteins. In addition, we provide succinct discussions on the subsequent applications of such hybrid systems and also give a brief overview of the rapidly progressing field of porphyrin-antibody conjugates. Since nucleic acid and peptide systems vary in structure, connectivity, functional group availability and placement, as well as stability and solubility, tailored synthetic approaches are needed for conjugating to each of these biomacromolecule types. In terms of tethering to ONs, porphyrins are typically attached by employing bioorthogonal chemistry (e.g., using phosphoramidites) that drive solid-phase ON synthesis or by conducting post-synthesis modifications and subsequent reactions (such as amide couplings, hydrazide-carbonyl reactions, and click chemistry). In contrast, peptides and proteins are typically conjugated to porphyrinoids using their native functional groups, especially the thiol and amine side chains. However, bioorthogonal reactions (e.g., Staudinger ligations, and copper or strain promoted alkyne-azide cycloadditions) that utilize *de novo* introduced functional groups onto peptides/proteins have seen vigorous development, especially for site-specific peptide-porphyrin tethering. While the ON-porphyrin conjugates have largely been explored for programmed nanostructure self-assembly and artificial light-harvesting applications, there are some reports of ON-porphyrin systems targeting clinically translational applications (e.g., antimicrobial biomaterials and site-specific nucleic acid cleavage). Conjugates of porphyrins with proteinaceous moieties, on the other hand, have been predominantly used for therapeutic and diagnostic applications (especially in photodynamic therapy, photodynamic antimicrobial chemotherapy, and photothermal therapy). The advancement of the field of porphyrinoid-bioconjugation chemistry from basic academic research to more clinically targeted applications require continuous fine-tuning in terms of synthetic strategies and hence there will continue to be much exciting work on porphyrinoid-biomacromolecule conjugation.

## Introduction

Porphyrins and their general derivatives (porphyrinoids) are pyrrole containing macrocycles with elaborate pi-conjugation pathways that result in vibrant colors observable by the naked eye. Indeed, the green colors in plants are due to the absorption capacity of the porphyrinoid cholorophyll A and its degradation, in the Fall, leads to other chromophores becoming more visible as orange and brown hues. More than mere aesthetics, biology harnesses the unique photophysical and chemical properties of porphyrinoids to drive immensely important processes including photosynthesis, blood oxygenation, and substrate oxidation. In these processes, the pophyrinoid cores interact with and are properly sequestered by, protein macromolecules that tune the activity of the macrocycles. In addition to classical photophysical and photochemical research (such as electron transfer, solar cells, and artificial light-harvesting) (Hsiao et al., 1996; Aratani et al., 2009; Beletskaya et al., 2009; Tanaka and Osuka, 2015; Zhang and Ying, 2015), researchers have focused on promising biomedical applications where porphyrins serve as photosensitizers (O’Connor et al., 2009) in e.g., photodynamic therapy (PDT), photodynamic antimicrobial chemotherapy (PACT) (Liu et al., 2012; Dosselli et al., 2013; Pereira et al., 2014; Meng et al., 2015; Yuan et al., 2017), photothermal therapy (PTT) (Zou et al., 2017), and as chromophores for biological imaging (Sibrian-Vazquez et al., 2005; Giuntini et al., 2011; Dondi et al., 2016).

From the abovementioned therapeutic modalities, PDT has been the most investigated and is used in the clinic to treat various cancers (including tumors of the esophagus, skin, head, neck, bladder, and lung). The reason for the adoption of PDT in the clinic is because it is generally safe and has few side effects. In this method, a photosensitizer first localizes in specific tumor tissue after intravenous injection. Subsequent irradiation of low-energy, tissue penetrating, light of appropriate wavelength (typically red light) leads to the activation of the photosensitizer from its ground state (S_0_) to the first excited singlet state (S_1_). The S_1_ state can release its energy by various pathways including emission of light as fluorescence or radiation-less transition. For PDT applications, an intersystem crossing takes place, transferring energy from the S_1_ state to the longer lived triplet state (T_1_). Population of the T_1_ state can lead to two different pathways of creating reactive oxygen species (ROS) necessary to initiate cell death. Type I reactions involve hydrogen or electron transfer between the T_1_ photosensitizer and biomolecules creating reactive radicals and other ROS. Type II reactions function by conversion of the ground-state triplet oxygen (^3^O_2_) to its reactive singlet state (^1^O_2_). Singlet oxygen leads to tissue destruction and apoptosis (O’Connor et al., 2009).

The other photosensitizer-based therapeutic modalities are newer technologies but are also promising. For example, the purpose of PACT is to treat infections with the ability to also overcome multidrug resistance. This modality follows the same principle of PDT in terms of production of ROS that are lethal to microbial pathogens (Meng et al., 2015). On the other hand, PTT facilitates tumor cell death by a thermal ablation mechanism, where the photosensitizer (i.e., photothermal agent) is designed to produce heat energy upon light irradiation (instead of fluorescence emission and production of singlet oxygen species). Importantly, these photothermal agents can also have applications as contrast agents for photoacoustic imaging (which has deeper penetration in biological tissues) (Zou et al., 2017).

In terms of photosensitizers being investigated for these biomedical applications, porphyrinoids have been used the most owing to their unique properties. These include their ability to efficiently absorb visible red light (high extinction coefficients), their long T_1_ state lifetimes (providing time to form ROS species and in particular generation of ^1^O_2_ with high yield), facile synthetic functionalization, structural diversity, and minimal dark toxicity (Kou et al., 2017; Lin et al., 2020). In many of the photosensitizer-based biomedical applications, the typically hydrophobic porphyrinoids need to be 1) solubilized in water, 2) inhibited from aggregation, and 3) also are required to localize selectively in target cells and tissue (Hamblin and Mroz, 2008). The judicious conjugation of porphyrins to biomacromolecules can address these issues. Further, porphyrin-biomacromolecule conjugation, in tandem with synthetic manipulation of the porphyrin peripheral substituents and metalation state, can result in modulation of key porphyrin features needed for biomedical applications (e.g., multiple absorption bands, emission near the far-red region of the visible electromagnetic spectrum, good photostability, and low dark toxicity).

**SCHEME 1:**
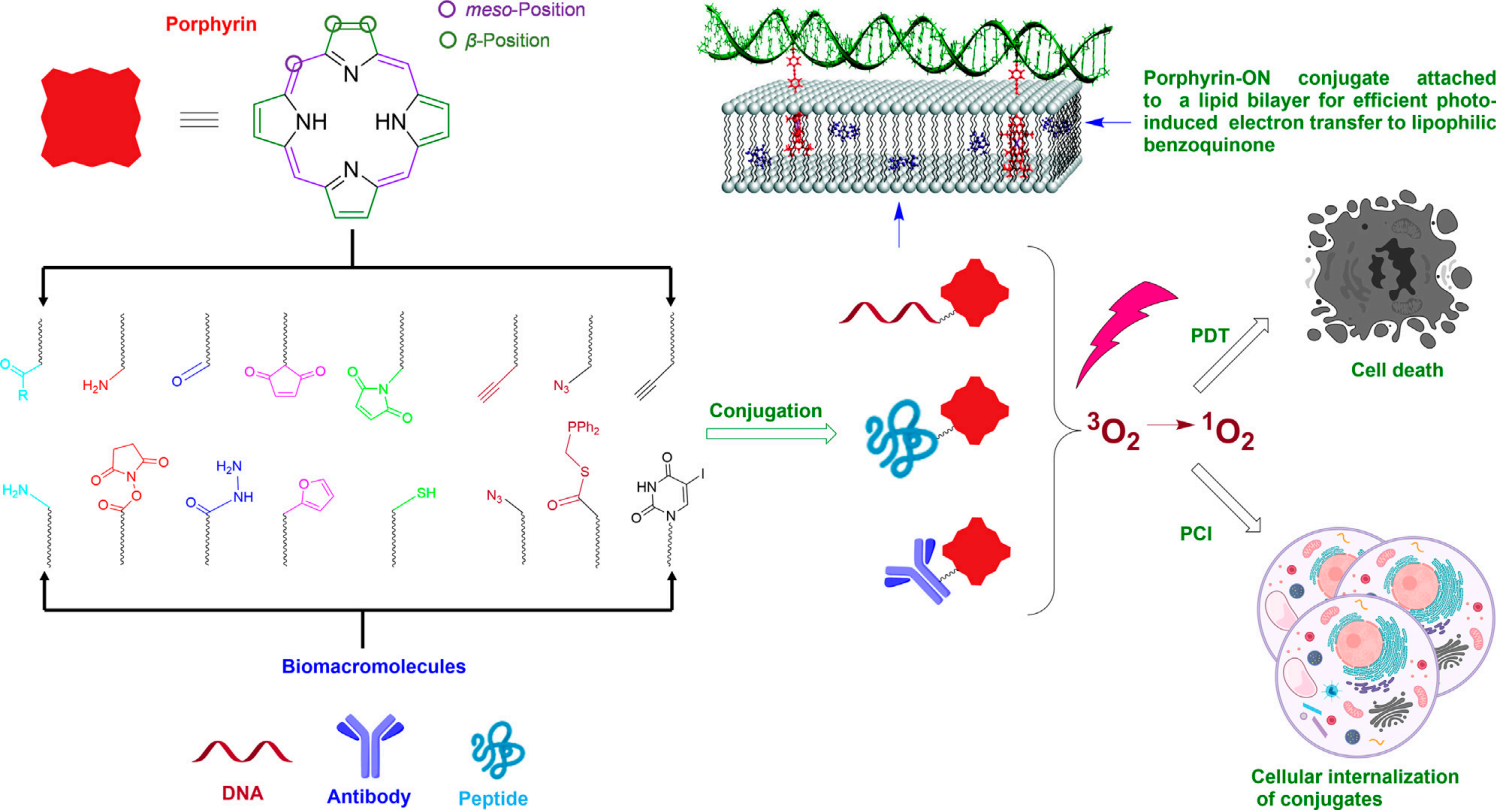
Schematic of attaching porphyrins to biomacromolecules using naturally occurring functional groups (thiol, amine) or bioorthogonal pairs and some applications of the conjugates. Note: Shown on the top left is a generalized porphyrin core. The circled β-and *meso*-positions on the porphyrin are where the majority of chemical functionalization is conducted for conjugation to biomacromolecules. A portion of this Scheme is adapted with permission from (Börjesson et al., 2010). The scheme was generated, in part, by using Biorender.com.

This review will focus on the novel methods developed and key applications that are uncovered by covalently linking porphyrinoids to three main classes of biomacromolecules: oligonucleotides (ONs), peptides and antibodies. These biomolecules are highly complex and contain multiple reactive functional groups and thus the methods used to selectively attach porphyrins to targeted sites on these biomacromolecules are also instructive for the general tethering of functional entities onto such biomolecules. In addition to utilizing naturally occurring reactive groups (e.g., amines and thiols), this manuscript will also discuss key bioorthogonal reactions that can be employed to facilitate porphyrin-biomacromolecule conjugation (Table 1). The bioorthogonal reactions are judiciously designed, with customized reactive partners, to be highly selective and do not readily react with other biological/chemical entities present in living cells/systems under physiological conditions (ambient temperature and pressure, neutral pH, and aqueous media). Further, bioorthogonal reactions, in general, should meet the criteria of kinetic-, thermodynamic-, and metabolic-stability, without generating materials toxic to living systems (Lang and Chin, 2014; Devaraj, 2018). Scheme 1 illustrates some common functionalities (naturally present or synthetically modified) in porphyrinoids as well as ONs and peptides that have been utilized to generate porphyrin-biomacromolecule conjugates. The scheme also shows some subsequent biomedical and light-harvesting applications that can be achieved. The following sections will expound on these conjugation chemistries and applications. Also, it should be noted that the focus herein will be on covalent tethering. Non-covalent interactions of porphyrinoids with biomacromolecules is also an area of active research. For example, porphyrinoids binding to peptides and proteins have been used for light harvesting applications, whilst porphyrin-DNA interactions (especially using cationic porphyrinoids) have applications ranging from detecting specific nucleic acid secondary structures, to nuclei staining, and as anti-microbial chemotherapeutic agents. For some pertinent reviews on this topic see; (Fiel, 1989; Schneider et al., 2002; Chakraborti, 2003; Zhu et al., 2016; Dognini et al., 2021; Mathew and Sujatha, 2021).

**TABLE 1:** Strategies for conjugation of porphyrinoids to key biomacromolecules.

*Porphyrin-biomacromolecule conjugations*			References
ON conjugation	Phosphoramidite chemistry	Attachment of porphyrinoids to sugar ring	Morales-Rojas and Kool (2002)
		Attachment of porphyrinoids to nucleobase moiety	Sessler et al. (2006)
		Porphyrinoid phosphoramidites without nucleoside	Vybornyi et al. (2014)
	Post-synthesis conjugation	Amide coupling	Casas et al. (1993), Pathak et al. (2019)
		Hydrazide reaction	Boutorine et al. (1996)
		Cooper catalyzed alkyne-azide cycloaddition (CuAAC)	Wellner and Wagenknecht (2014)
		Diels-Alder cycloaddition	Wellner and Wagenknecht (2014)
Peptide conjugation	Bioorthogonal reactions	Staudinger ligation	Nilsson et al. (2000)
		Cooper catalyzed alkyne-azide cycloaddition (CuAAC)	Sokolova et al. (2013)
		Strain promoted alkyne-azide cycloaddition (SPAAC)	Dondi et al. (2016)
		Olefin metathesis	Delaude and Noels (2005)
	Ligation *via* cysteine residue	Thiol-maleimide ligation	Wang et al. (2012)
		Thiol-haloacetamide ligation	Chaloin et al. (2001)
	Ligation through amino group	Thiourea formation	Sibrian-Vazquez et al. (2005)
		Amide coupling	Solladié et al. (2001)
Antibody conjugation	Thiol-maleimide ligation		Hemming et al. (1993)
	Hydrazide reaction		Goff et al. (1994)
	Amide coupling		Gordon et al. (2015)
	Strain promoted alkyne-azide cycloaddition (SPAAC)		Maruani et al. (2015)
	Amine-isothiocyanate coupling		Hudson et al. (2005)
	SNAP-tag technology		Hussain et al. (2011)

## DNA-Porphyrin Conjugates

In terms of attaching functional systems onto ONs, the vast majority of work has been conducted on deoxyribonucleic acid (DNA) conjugation. An array of such DNA-small molecule chimeras have been prepared to incorporate, *inter alia*, drug candidates, polymers, vitamins, peptides, fluorophores, protein inhibitors, macrocyclic hosts, nanoparticles, and metal complexes onto DNA architecture (Nesterova et al., 2007; Singh et al., 2010; Hunger et al., 2014; Zhu et al., 2015; Lou et al., 2016; Zhou et al., 2018; Lueckerath et al., 2019; Pathak et al., 2019; Huang et al., 2020). Here, the high-fidelity self-assembly properties of DNA sequences offer a wide range of supramolecular design possibilities with numerous potential applications in the field of DNA nanotechnology, nucleic acid therapeutics, and DNA photonics (Sharma et al., 2014; Seeman and Sleiman, 2017; Kuzyk et al., 2018). Although many chemical reaction pathways can be adopted to functionalize ONs, few selected reactions are frequently executed for ON modification (Singh et al., 2010; Madsen and Gothelf, 2019). For DNA-porphyrin conjugates, there are two main routes for tethering porphyrin to ONs; 1) porphyrin-modified phosphoramidites, and 2) post-synthesis porphyrin conjugation. After porphyrin molecules are successfully attached to ONs, the first consequence is that the hydrophobic porphyrin dyes acquire significant aqueous solubility, can interact with the tethered nucleobases, and depending on the interaction, can demonstrate chiral behavior (induced by DNA helicity). Besides, the secondary structures formed by DNA self-assembly can provide the porphyrin dyes with a unique surrounding microenvironment and also act as a programmed template for the precise arrangement of these dye molecules.

### Porphyrin-Modified Phosphoramidites

**SCHEME 2:**
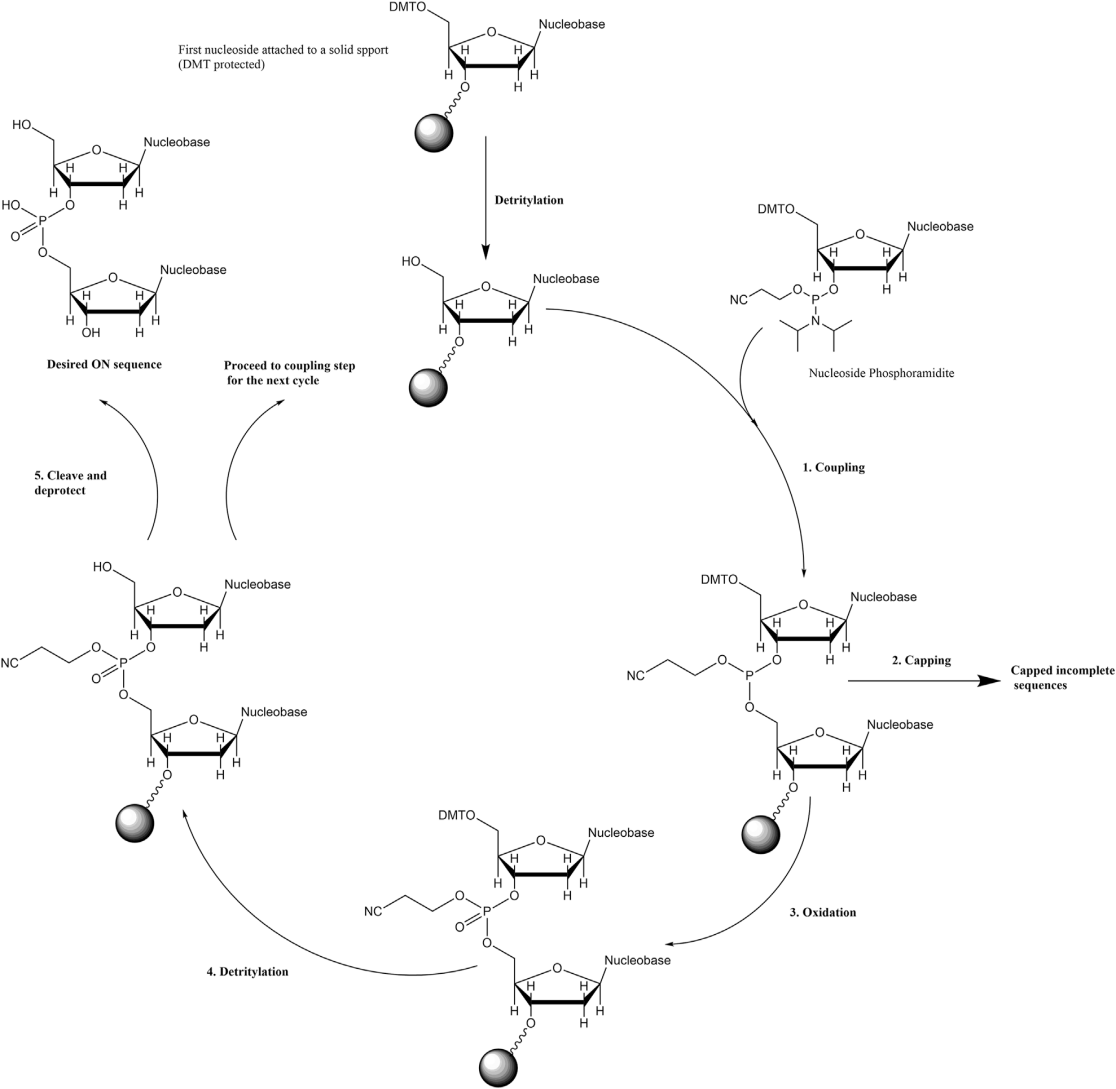
General scheme for solid-phase phosphoramidite-based (3′ to 5′) ON synthesis.

Phosphoramidite-based solid-phase synthesis is one of the dominant methods employed to tether porphyrins onto ONs. In general, this method starts with attaching the 3′ end of a 5′-dimethoxytrityl (DMT)-protected nucleoside onto a resin *via* a custom linker (Scheme 2). Then the stepwise coupling of nucleoside phosphoramidites to the growing sequence is carried out by deprotecting the acid-labile DMT group (i.e., detritylation) on the terminal nucleoside prior to the addition of each incoming phosphoramidite. For the coupling step, a tetrazole-based acidic catalyst (e.g., 5-(ethylthio)-1H-tetrazole) activates the incoming phosphoramidite by protonating the diisopropyl amino group, followed by the attack by the 5′-OH group (of the ON sequence on the solid support) onto the phosphorous atom to displace the diisopropyl amine. Capping off the incomplete sequences to mask the free -OH groups and oxidation of phosphorous (III) to phosphorous (V) are important steps before detritylation and the next coupling cycle. Finally, deprotection under basic conditions cleaves the ON sequences from the solid support, removes any protecting group from the exocyclic amines of nucleobases, and releases the 2-cyanoethyl protecting groups on the phosphates. The porphyrin phosphoramidite building blocks are generally prepared by attaching porphyrins to the sugar rings or to the nucleobase units. These phosphoramidites go through similar cycles of solid-phase ON synthesis to offer porphyrin-ON conjugates.

#### Porphyrins Directly Attached to Sugar Rings

**FIGURE 1:**
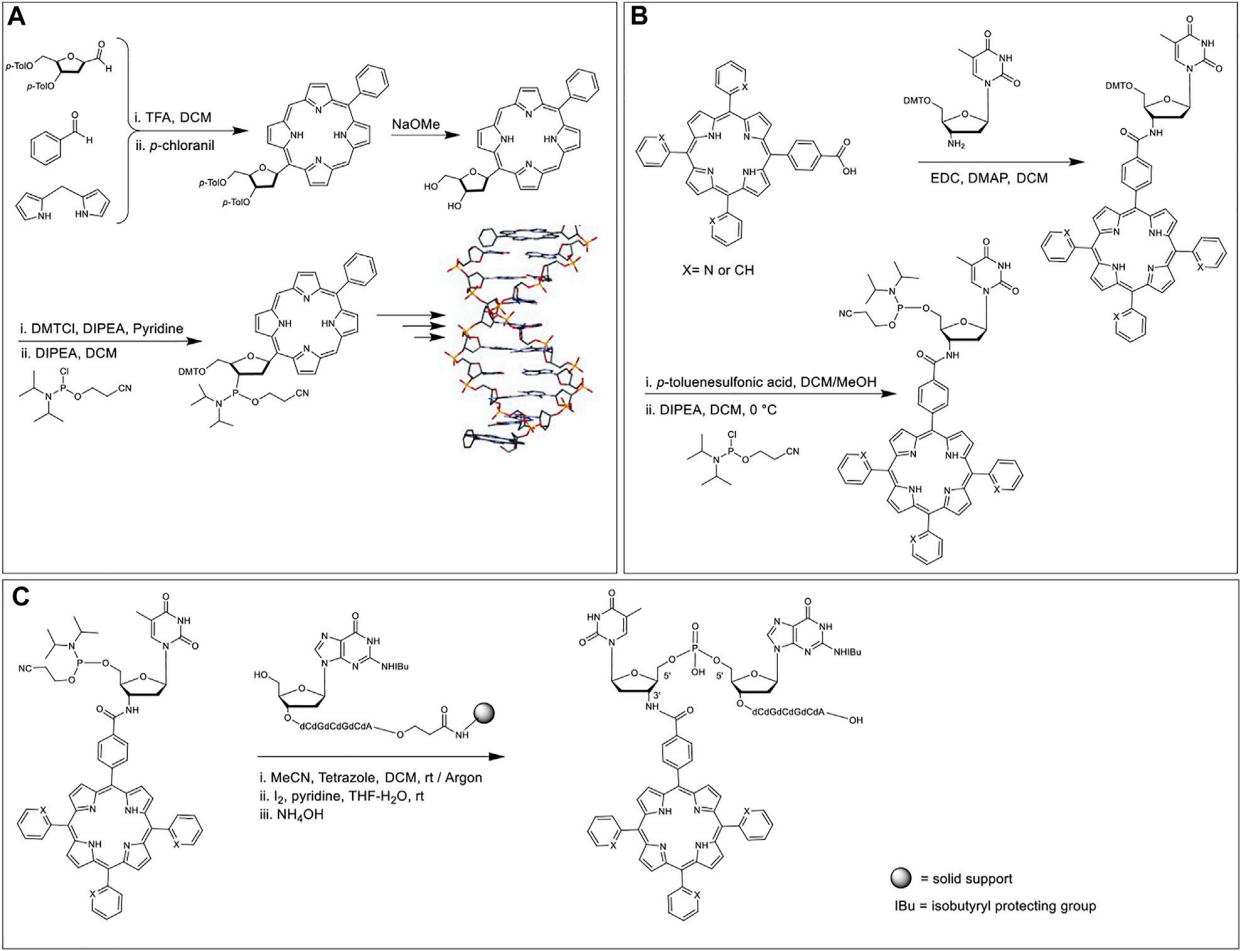
**(A)** Porphyrin phosphoramidite as a nucleobase replacement synthesized in a multistep reaction scheme. Hybridization of self-complementary strands containing porphyrin units affords a DNA duplex where the porphyrin chromophores interact with the terminal nucleobases. Adapted with permission from (Morales-Rojas and Kool, 2002). **(B)** Amide coupling between 3′-amino deoxyribose thymidine nucleoside and porphyrin monocarboxylic acid, subsequent DMT deprotection, and phosphitylation affords the requisite 3′-porphyrin phosphoramidite. **(C)** An atypical 5′ to 5′ coupling using 3′-porphyrin-5′-phosphoramidite, followed by oxidation and deprotection (Balaz et al., 2005).

Porphyrin-modified phosphoramidites as nucleoside phosphoramidite analogs have been achieved by attaching porphyrins to the sugar rings at 1′ carbon as a total replacement of the nucleobase unit (Morales-Rojas and Kool, 2002) or by tethering the porphyrin to the nucleoside phosphoramidite *via* amino functionalization of 3′ sugar carbon (Balaz et al., 2005). In regard to the former strategy, in 2002, Kool et al. (Morales-Rojas and Kool, 2002) synthesized a porphyrin nucleoside by reacting 3′,5′-bis-*O*-toluoyl-protected deoxyribose-C1-carboxaldehyde with benzaldehyde and dipyrromethane under Lindsey-like condensation conditions (Lindsey et al., 1987) (i.e., condensation of aldehydes and pyrroles in the presence of strong acid catalysts and subsequent oxidation) followed by the deprotection of the toluoyl groups. Selective 5′-*O*-tritylation and 3′-*O*-phosphitylation afforded the requisite 2-cyanoethyl phosphoramidite monomer (Figure 1A). This porphyrin phosphoramidite was introduced onto the growing ON sequence at either the terminal positions or the interior backbone. Duplex structures formed by a pair of self-complementary ON strands modified with porphyrin macrocycles at the termini demonstrated higher thermal stability (∆*T*_m_ = 14.7°C) as compared to the unmodified congener. End stacking, *via* aromatic interactions, of porphyrins with the terminal nucleobases was posited to be the reason for the enhanced stabilization of the double helix. In contrast, internally tethered porphyrins (attached to the phosphate backbone) of the DNA sequence intercalate within the duplex and lead to a slight destabilization of the double helix in comparison to the perfectly complementary analog. Interestingly, when a sequence with a nucleobase mismatch was used, the internal porphyrin modification led to the enhancement of duplex stability (versus the unmodified control).

In 2005, Balaz et al. synthesized 3′-tetraarylporphyrin-thymidine-5′-phosphoramidites and used them as building blocks in the synthesis of ON–porphyrin conjugates (Balaz et al., 2005). It was reportedly the first example of porphyrins incorporated onto the 3′ position of ONs. *Meso*-trispyridylphenylporphyrin carboxylic acid and *meso*-tetraphenylporphyrin carboxylic acid were coupled with 3′-amino-5′-dimethoxytritylthymidine using 1-Ethyl-3-(3-dimethylaminopropyl)carbodiimide (EDC) and 4-Dimethylaminopyridine (DMAP) as coupling reagents (Figure 1B). The DMT group was removed with a 4% solution of *p*-toluenesulfonic acid followed by the reaction with 2-cyanoethyl-*N,N*-diisopropylchloro phosphoramidite to offer the desired 3′-porphyrin-thymidine 5′-phosphoramidites. Two separate self-complementary sequences, 5′-ACGCGCGT-3′ and 3′-T-5′-GCGCGCA-3′ were chosen for the study where the terminal thymidine residues bear the porphyrin moiety. The former sequence was synthesized *via* automated DNA synthesis in the reverse 5′ to 3′ direction. The latter sequence was synthesized in the typical 3′–5′ direction up to the penultimate 5′-guanosine residue, after which, the terminal porphyrin-thymidine phosphoramidite was introduced *via* a 5′–5′ coupling (Figure 1C).

#### Porphyrins Attached to the Nucleobase Moiety

Palladium catalyzed cross coupling is an attractive method to attach porphyrins off the nucleobase units since both partners are aromatic. Indeed, over the decades, there have been numerous reports of utilizing Palladium catalyzed coupling to generate porphyrinoid- and pyrrole-nucleobase conjugates illustrating the broad scope of these reactions to create new C-C bonds between nucleobases and porphyrin-related moieties (Sessler et al., 2006; Torres et al., 2007; Pierce et al., 2008). In particular, Sonogashira coupling between alkyne-modified porphyrins and iodo-modified uridine nucleosides are frequently employed by researchers to generate porphyrin-based phosphoramidites.

**FIGURE 2:**
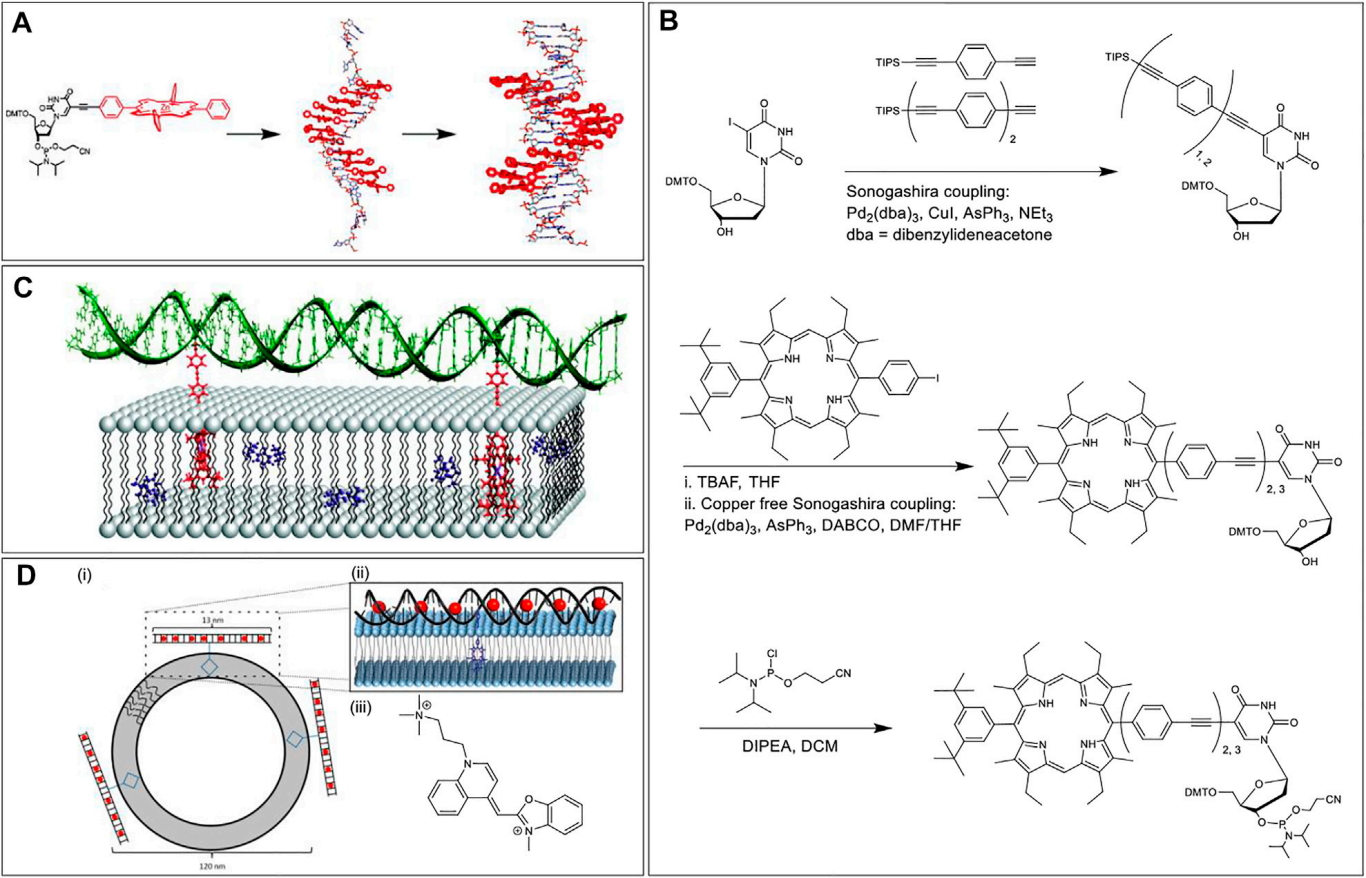
**(A)** Solid-phase automated DNA synthesis using a phosphoramidite-porphyrin nucleotide (prepared *via* Sonogashira coupling) as building blocks offers a DNA sequence with multiple porphyrin units in a row. The porphyrin units align themselves in the major groove when the sequence is hybridized with a complementary DNA sequence. Reprinted with permission from (Fendt et al., 2007). **(B)** Synthesis of the porphyrin nucleoside building block with 2 or 3 repeating units of phenylethynylene linkers. **(C)** Hybridization of DNA on the lipid surface anchored by two porphyrins embedded in the lipid bilayer. The length of the phenylethynylene linker was optimized to ensure proper assembly with the porphyrin chromophores placed inside the lipid layer and DNA duplex tangential to the membrane surface. Adapted with permission from (Börjesson et al., 2010). **(D)** (i) Duplex DNA conjugated to a central porphyrin that anchors the system to lipid vesicles. (ii) A zoomed-in view of YO dyes intercalated in the DNA duplex and porphyrin embedded in the lipid bilayer. (iii) Chemical structure of the YO dye. Reprinted with permission from (Woller et al., 2013).

The Stulz research group, in 2007, carried out Sonogashira coupling between 5-iodo deoxyuridine and alkyne functionalized tetraphenylporphyrin to synthesize a phosphoramidite porphyrin-nucleotide (Figure 2A) (Fendt et al., 2007). Subsequently, up to 11 tandem porphyrin units were covalently tethered to a DNA strand *via* standard solid-phase ON synthesis. After the conjugation and placement in aqueous media, hydrophobic stacking led to the porphyrin units interacting with each other resulting in the broadening of the absorption bands and quenching of the emission. Moreover, when complementary sequences were hybridized, the porphyrin moieties aligned themselves in the major groove of the double helix. Further, the chirality imparted by the helicity of the DNA domain resulted in a circular dichroism signal in the porphyrin Soret band region.

In 2010, Albinsson et al. used repetitive phenylethynylene linkers to tether a porphyrin moiety at a certain distance from a 39-mer DNA core (Börjesson et al., 2010). First, the researchers synthesized a deoxyuridine porphyrin nucleoside phosphoramidite *via* two Sonogashira coupling reactions. The first Sonogashira coupling to yield triisopropylsilyl ether (TIPS) protected phenylethynylene nucleoside involved tris(dibenzylideneacetone)dipalladium (0) as the Pd catalyst, CuI as a copper (I) cocatalyst, triphenylarsine (AsPh_3_) as a dative ligand, and triethylamine (NEt_3_) as a base as well as the reaction solvent. In the second copper-free Sonogashira coupling reaction, to generate porphyrin phenylethynylene nucleoside, the copper (I) cocatalyst was excluded to prevent Cu-metalation of the porphyrin core. In addition, the copper-free coupling was optimized by using 1,4-diazabicyclo [2.2.2]octane (DABCO) as the base and dimethylformamide (DMF)/tetrahydrofuran (THF) mixture as the reaction solvent (Figure 2B). After phosphitylation, the resultant phosphoramidite was then used as a building block to tether porphyrins to the DNA sequence followed by the zinc metalation of the porphyrin core. When the Zn porphyrin-DNA conjugate was mixed with liposomes dispersed in water, the hydrophobic effect kept the porphyrin embedded in the lipid bilayer, and the DNA domain stayed in the aqueous medium. Further hybridization with a complementary strand generated a duplex architecture on the lipid surface. More importantly, the use of doubly modified strands resulted in the generation of two porphyrin anchors that enables the DNA duplex to be oriented tangential to the membrane surface (Figure 2C). Importantly, binding of the porphyrins into the lipid bilayer protected the porphyrin from the bulk aqueous solution, precluded the hydrophobic stacking among porphyrin units, and made the system redox-active facilitating electron transfer between the excited-state porphyrin and a benzoquinone electron acceptor, placed within the bilayer.

Again in 2013, Albisson et al. used a similar DNA-Porphyrin-Lipid architecture to employ free-base porphyrin and YO-PRO-1 (YO) as acceptor and donor dyes, respectively, in a DNA-based FRET architecture (Woller et al., 2013). The YO dyes were intercalated in the DNA duplex with a spacing of ∼10 Å and upon excitation at 483 nm (where only the YO dyes absorb), the YO dyes transferred energy directionally to the porphyrin embedded in the lipid bilayer *via* FRET (Figure 2D). Prior to energy transfer to the porphyrin unit, the YO dyes transfer energy among themselves *via* homoFRET. In this artificial light-harvesting assembly, when the porphyrin emission was monitored at 700 nm, the antenna effect facilitated by the YO dyes was calculated to be 12 (with a 20:1 ratio of YO to porphyrin). This multi-component self-assembly clearly demonstrates how the programmability of DNA assembly, along with the use of other supramolecular interactions, can generate a well-defined light-harvesting antenna.

#### Porphyrin Phosphoramidites Without a Nucleoside Component

**FIGURE 3:**
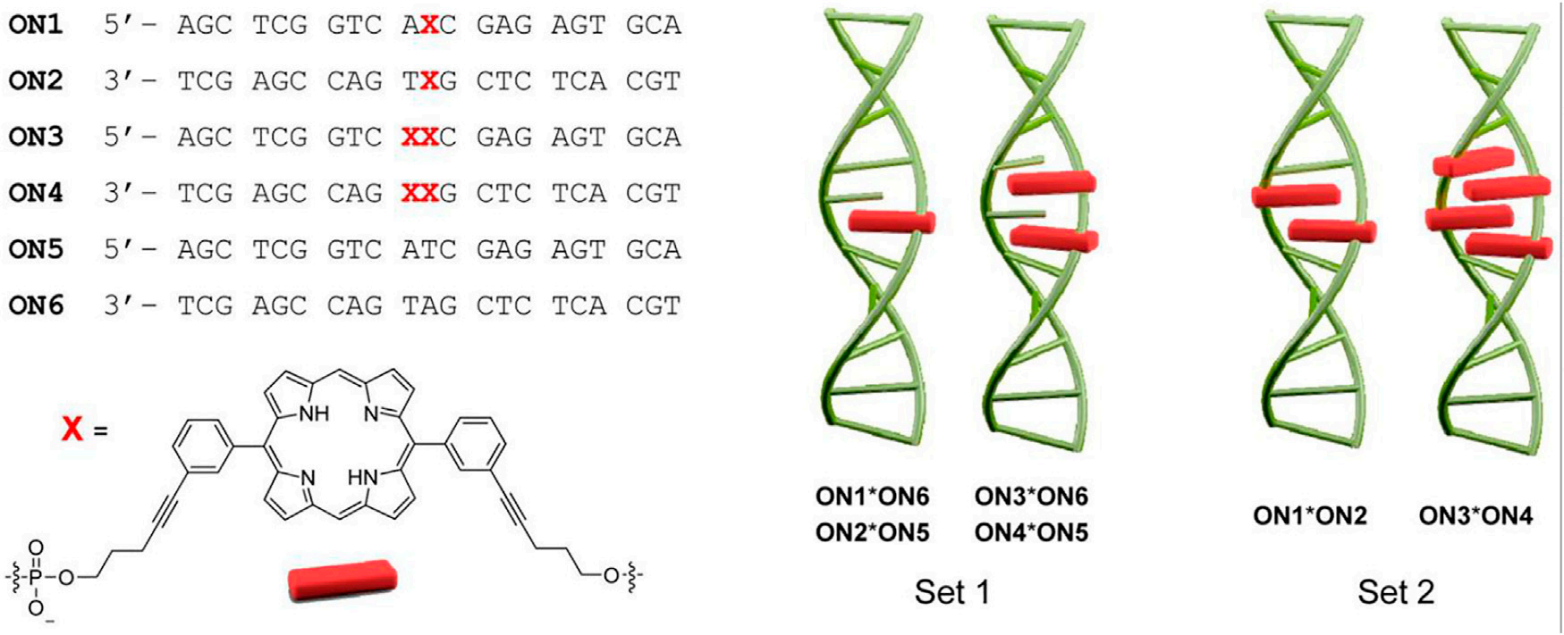
ON sequences modified with different numbers of the porphyrins (denoted by X) using phosphoramidite chemistry. The hybridization of the ONs enables the formation of various duplexes differing in the number of stacked porphyrins. Adapted with permission from (Vybornyi et al., 2014).

The Häner group in 2014 developed porphyrin phosphoramidites that would completely replace a nucleoside component at the specific site of attachment (Vybornyi et al., 2014). Specifically, a 5,15-bisphenyl-substituted porphyrin was modified such that one arm of the porphyrin possessed a phosphoramidite unit, and the other arm bore a DMT-protected hydroxyl group. Using the solid-phase synthesis method, ON sequences (ON1 through ON6) bearing 0, 1, or 2 porphyrin units were synthesized. ON1, ON3, and ON5 represent the same sequence except for the sites where nucleobases are substituted by the porphyrins. These ONs (ON1-ON3) are complementary to another set of ONs with the same sequence (ON2, ON4, and ON6). Hybridization of the complementary sequences generated duplexes with up to 4 porphyrins next to each other (Figure 3). Compared to the unmodified duplexes the substitution of nucleobases with the porphyrin units resulted in the reduction of the melting point (up to 12°C per modification). Further, the resulting H-aggregation of the porphyrins led to fluorescence quenching.

### Post-Synthesis Porphyrin Conjugation

While porphyrin phosphoramidite precursors can be readily obtained for solid-phase incorporation of porphyrins onto ONs at any position, the chemistry can be tedious in the sense that each porphyrin has to be derivatized with a phosphoramidite and with protected hydroxyl moieties. An alternative approach is to pursue a post-ON-synthesis modification strategy that delivers the desired porphyrin-modified ONs *via* more common organic reactions. The post-synthesis modification of ONs also allows porphyrins to be attached directly to the nucleobases or at other terminal or interior positions *via* functionalized linkers.

#### Amide Coupling

**FIGURE 4:**
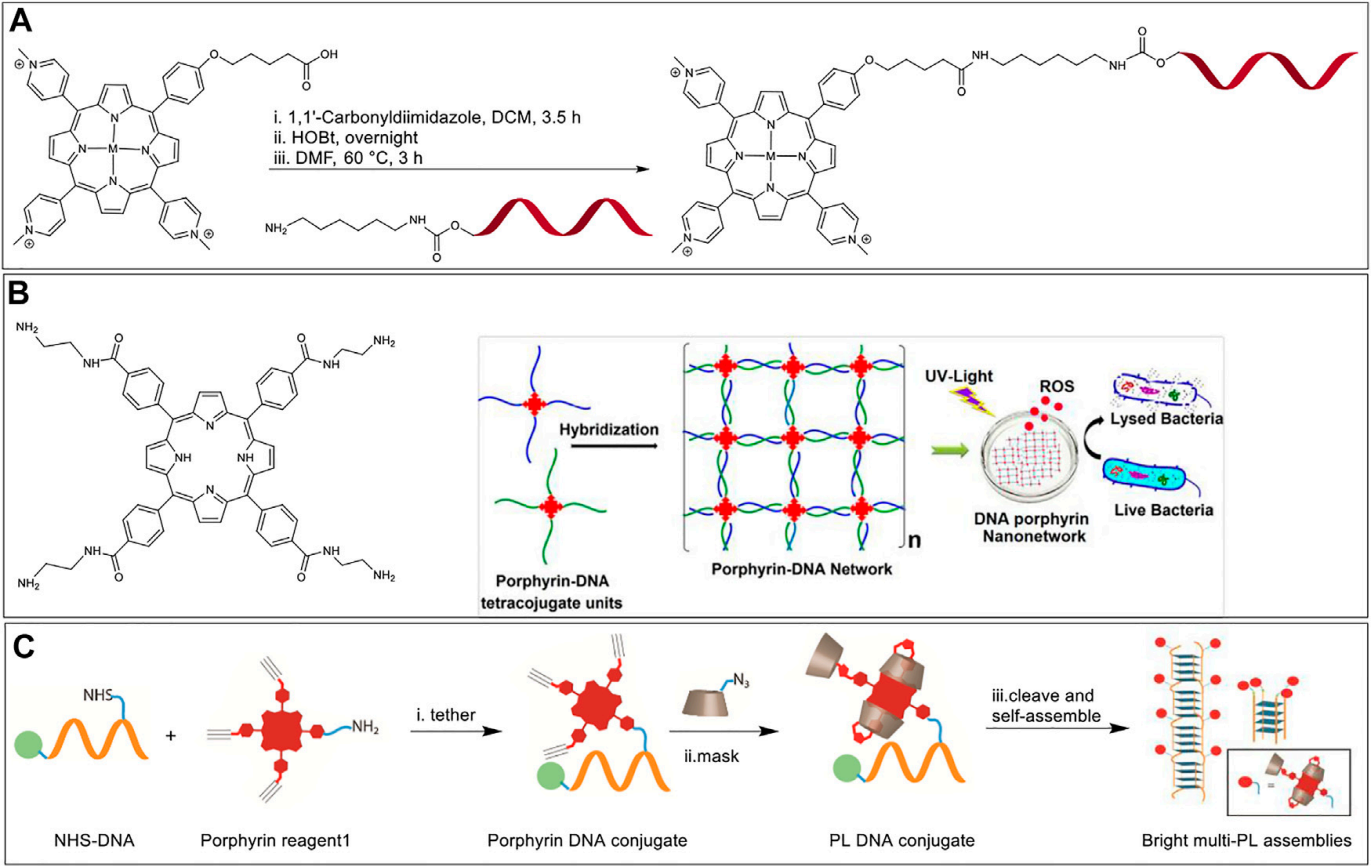
**(A)** Synthesis of hybrid-metalloporphyrin-DNA conjugates from the reaction of 5′-amino-modified DNA and a benzotriazole activated porphyrin. M = Mn (III)-axial ligand, Ni (II), Cu (II), Co (II). DNA sequence = T17 (Casas et al., 1993). **(B)** Left: A tetra amine functionalized porphyrin macrocycle; Right: A pair of porphyrin-DNA hybrids tetrasubstituted with complementary DNA arms that self-assemble to form an ordered network. When excited with UV light the nanonetwork produces ROS and exhibits antibacterial activity. Adapted with permission from (Kumari et al., 2017). **(C)** A bottom-up approach to develop G-quadruplex-based fluorescent nanostructures that involves (i) synthesis of a porphyrin-DNA hybrid *via* NHS ester-amine coupling, (ii) masking the porphyrin with PMβCD hosts *via* CuAAC, and (iii) self-assembly of the final conjugate.

The reaction of an activated carboxylic acid with an amine generates a stable amide bond and is referred to as amide (or peptide) coupling. Carboxylic acids can be activated *in situ* by reacting with carbodiimides (e.g., dicyclohexylcarbodiimide, DCC) and aminium/phosphonium derivatives of benzotriazole (e.g., HATU, BOP) or can be first transformed to storable activated esters (e.g., *N*-hydroxysuccinimido esters). An early example of a Porphyrin-DNA conjugate was reported by Meunier et al., in 1993 who used amide bond-formation (Casas et al., 1993). In this paper, the researchers conjugated a cationic manganese (III) porphyrin (Mn-TMPyP) that is also appended with a carboxylic acid functional group onto ONs at the 5′ position. For this, they activated the carboxylic acid functional group with 1-1′-carbonyldiimidazole followed by the reaction with 1-hydroxybenzotriazole (HOBt). Unmodified ON sequences do not have primary aliphatic amines that react efficiently with the activated porphyrin ester. Hence, a 5′-amino-modified ON sequence with a C6 linker was used to react with the activated porphyrin to afford the porphyrin-DNA conjugate (Figure 4A). In terms of function, the cationic Mn-TMPyP acts as an endonuclease to hydroxylate C-H bonds of DNA sugars at low concentrations. Hence, when hybridized to the AUG initiation codon of the HIV-1 TAT gene, the porphyrin-DNA conjugates were able to cleave the target sequence showing promise as a potential antiviral agent.

In 2017, Das et al. (Kumari et al., 2017) and Campidelli et al. (Chatelain et al., 2017) independently reported the self-assembly of multi-branched Porphyrin-DNA hybrid molecules into elaborate nanostructures. In both manuscripts *meso*-tetra(4-carboxyphenyl)porphyrin was functionalized, with 4 single-stranded ON sequences generating a porphyrin molecule linked covalently with four ON arms. To achieve tetra-ON-porphyrin hybrids, Campidelli et al. first modified *meso*-tetra(4-carboxyphenyl)porphyrin to a tetra azido derivative *via* amide coupling with a 2-azidoethanamine linker, followed by copper catalyzed alkyne-azide cycloaddition (CuAAC) reaction with alkyne modified ONs, whereas Das et al. first modified *meso*-tetra (4-carboxyphenyl) porphyrin to a tetra amino derivative, followed by a reaction with imidazole activated 5′-phosphate group on the ONs. Incubation with another similar tetra substituted porphyrin-DNA hybrid with complementary sequences facilitated the formation of extensive nanostructure networks (Figure 4B). In the specific work of Das et al. the porphyrin-DNA hybrid nanostructures were shown to exhibit specific antibacterial activity against Gram-positive bacteria, *Staphylococcus aureus,* when irradiated with white light due to generation of reactive oxygen species (ROS). The conversion of non-fluorescent dihydrorhodamine to fluorescent rhodamine was monitored to measure the production of ROS. The viability of growing bacterial cultures was measured (in colony forming units, CFU) with free porphyrin and porphyrin nanonetwork in light and dark conditions. The percentage CFU count was above 90% for all culture samples in dark. However, the percentage CFU count dropped to 10 ± 4%, and 2 ± 1.5% in the cultures mixed with free porphyrin and porphyrin nanonetwork, respectively when exposed to white light (100 W bulb) for 30 min. From these studies the authors concluded that the self-assembled porphyrin nanonetwork generates more ROS as compared to the free porphyrin system, and therefore is more effective in killing the bacteria. Here, the uniform dispersion of the porphyrin moieties separated by negatively charged DNA duplexes was believed to preclude the self-quenching of the porphyrin units thereby enhancing the efficiency of ROS generation.

In 2019, Pathak et al. used a guanine quadruplex (G-quadruplex) scaffold to generate nanostructures capable of arraying multiple porphyrins in close spacing (Pathak et al., 2019). In particular, a short G-rich sequence (G_4_T_2_G_4_) was modified with a single porphyrin unit at an interior thymidine residue *via* coupling of an ON-appended *N*-hydroxy succinimidyl (NHS) ester and an amino porphyrin (Figure 4C). Next, three mono-azido per-*O*-methylated β-cyclodextrin (PMβCD) hosts were covalently attached to the remaining porphyrin arms *via* CuAAC. The PMβCD hosts hydrophobically encapsulate the porphyrin core, thereby masking the chromophore from the aqueous environment which was shown to be essential to preclude dye aggregation and fluorescence quenching. In addition, the intramolecular encapsulation also kept the porphyrin from disrupting the secondary structure of the DNA self-assembly. The self-assembly of the ON sequence resulted in directional G-quadruplex nanowires decorated with hundreds of bright porphyrin dyes with 1–2 nm spacing. These masked porphyrin assemblies were upto 180-fold brighter than the analogs lacking the PMβCD encapsulation. It is of note that Hamilton and colleagues in 2007 employed similar amine-NHS ester coupling to tether amino-functionalized porphyrin with NHS-ester modified G-rich ON sequences. Their work involved an investigation of the effect of porphyrin stacking interactions in the modulation of G-quadruplex assembly and stability (Jayawickramarajah et al., 2007).

#### Hydrazide Reaction

Another highly selective reaction that can be conducted post ON-synthesis involves the reaction between hydrazides and carbonyl compounds to generate hydrazones. The hydrazide to hydrazone transformation can typically be considered as a bioorthogonal reaction as carbonyl compounds and hydrazides react selectively in a biological milieu without interfering with other biological metabolites. However, caution has to be applied to ensure the naturally occurring aldehydes/ketones and other highly reactive electrophiles are in low concentration to avoid undesired side reactions.

**FIGURE 5:**
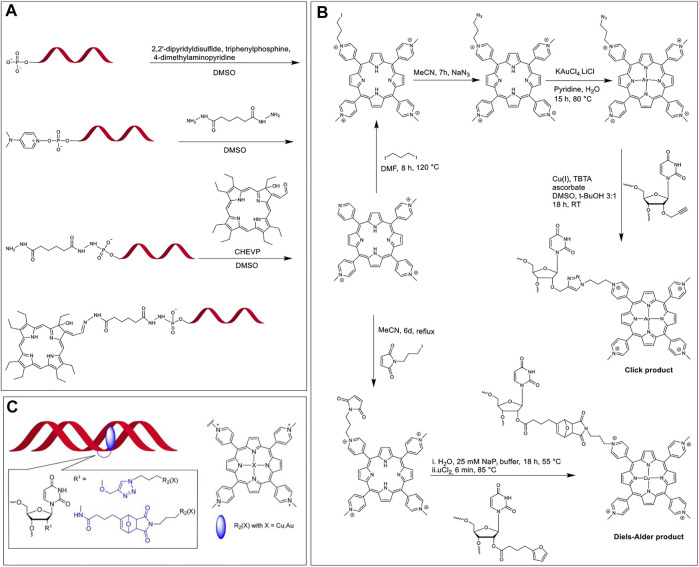
**(A)** Activation of the 5′-phosphate group of ON and modification with a dihydrazide linker, followed by the coupling of aldehyde bearing CHEVP with hydrazide modified ON (Boutorine et al., 1996); **(B)** Synthesis of modified TMPP in a multi-step reaction scheme followed by the post-synthetic modification of ON *via* CuAAC and Diels-Alder cycloadditions; **(C)** TMPP (blue) tethered at the 2′-position of modified uridine intercalating with the DNA duplex. Note; the linker structures are highlighted in blue in the inset. Adapted with permission from (Wellner and Wagenknecht, 2014).

In 1996, Hélène et al. investigated the site-specific DNA damage of single-stranded, double-stranded, and triplex nucleic acids by tethering chlorins to corresponding DNA sequences followed by irradiation (Boutorine et al., 1996). Chlorins are porphyrin derivatives wherein one of the β,β′-pyrrolic double bonds are saturated, resulting, *inter alia*, in modulated optical and redox properties (Taniguchi and Lindsey, 2017). In this system, the DNA sequence provided target specificity with the chlorin acting as a photosensitizer. The researchers synthesized a chlorin-type photosensitizer, CHEVP (chlorin derived from heptaethylvinylporphyrin) by photochemical conversion of the corresponding vinyl porphyrin precursors. Next, the 5′-phosphate of the ON was activated by reacting with 2,2′-dipyridyldisulfide, triphenylphosphine, and 4-dimethylaminopyridine to yield 4-dimethylaminopyridinium derivative of the ON (Figure 5A). The activated ON was reacted with an adipic acid dihydrazide linker to obtain a hydrazide functional group at the terminal end. Finally, the aldehyde on the CHEVP moiety was reacted with the hydrazide functionalized ON to offer the required ON-chlorin conjugate. The ON-CHEVP (sequence: 5′-TTC​TTC​TCC​TTT​CT-3′) was incubated with a single strand, duplex, and hairpin loop structures to form a duplex and two triplex structures. When these hybrid assemblies were irradiated using 668 nm light under aerobic conditions, site-specific DNA cleavage at guanine positions was observed due to singlet oxygen generation by the photosensitized chlorin. As piperidine-labile photo-adducts were formed due to oxidative damage to guanine sites, the DNA cleavage was more pronounced when the samples were treated with 1 M piperidine.

#### Copper Catalyzed Alkyne-Azide Cycloaddition and Diels-Alder Cycloaddition

Copper catalyzed click reaction between an alkyne and azide functionalities has revolutionized the way how chemical species are connected. This reaction is facile and can be performed under ambient aqueous conditions. In this reaction, terminal azide and alkyne groups react with each other and the major product is typically the 1,4-disubstituted 1,2,3-triazoles. Since alkyne and azide functional groups are uncommon in natural systems and the reaction proceeds fast to generate a stable triazole ring, this reaction is bioorthogonal. However, one challenge posed by CuAAC, especially for *in vivo* studies, is the toxicity of the Cu catalyst. To circumvent this issue, numerous copper-free versions of alkyne-azide click chemistry (e.g., strain promoted cycloadditions) have been developed for more biocompatible applications. Another popular reaction for tethering two components is the Diels-Alder cycloaddition, which is a pericyclic reaction between an alkene and a conjugated diene to yield a six-membered ring.

In 2014, Wellner and Wagenknecht synthesized porphyrin-ON conjugates *via* post-synthetic modification using both CuAAC and Diels-Alder cycloaddition reactions (Wellner and Wagenknecht, 2014). The team developed a uridine phosphoramidite functionalized at the 2’ ribose OH with either an alkyne group for post-synthesis CuAAC or a furan moiety for post-synthesis Diels-Alder cycloaddition, and thus achieved ON sequences with internal uridine modifications. They also functionalized *meso*-tetra-(4-*N*-methylpyridyl)porphyrin (TMPP) with azide and maleimide units for CuAAC and Diels-Alder cycloaddition, respectively. As seen in Figure 5B, CuAAC generates a simple triazole linker whereas, the Diels-Alder cycloaddition results in a linker with a tricyclic portion. These two linkers had a significant effect on the interaction between the ON backbone and TMPP. When the porphyrin-ON conjugates were hybridized with their complementary sequences to form DNA duplexes (Figure 5C), the bathochromic shift in the Soret and Q bands of the porphyrin units in UV-vis spectra along with the presence of a prominent negative peak at the porphyrin Soret region in CD spectra were considered to be the signature of intercalation of the porphyrin units in the DNA duplex. In contrast to the CuAAC product, in the tricyclic Diels-Alder product, the intercalation of TMPP in the DNA duplex was hindered (due to steric restrictions), which was evidenced by the absence of the bathochromic shift of the Soret peak and a significantly diminished CD peak, as compared to CuAAC product.

**FIGURE 6:**
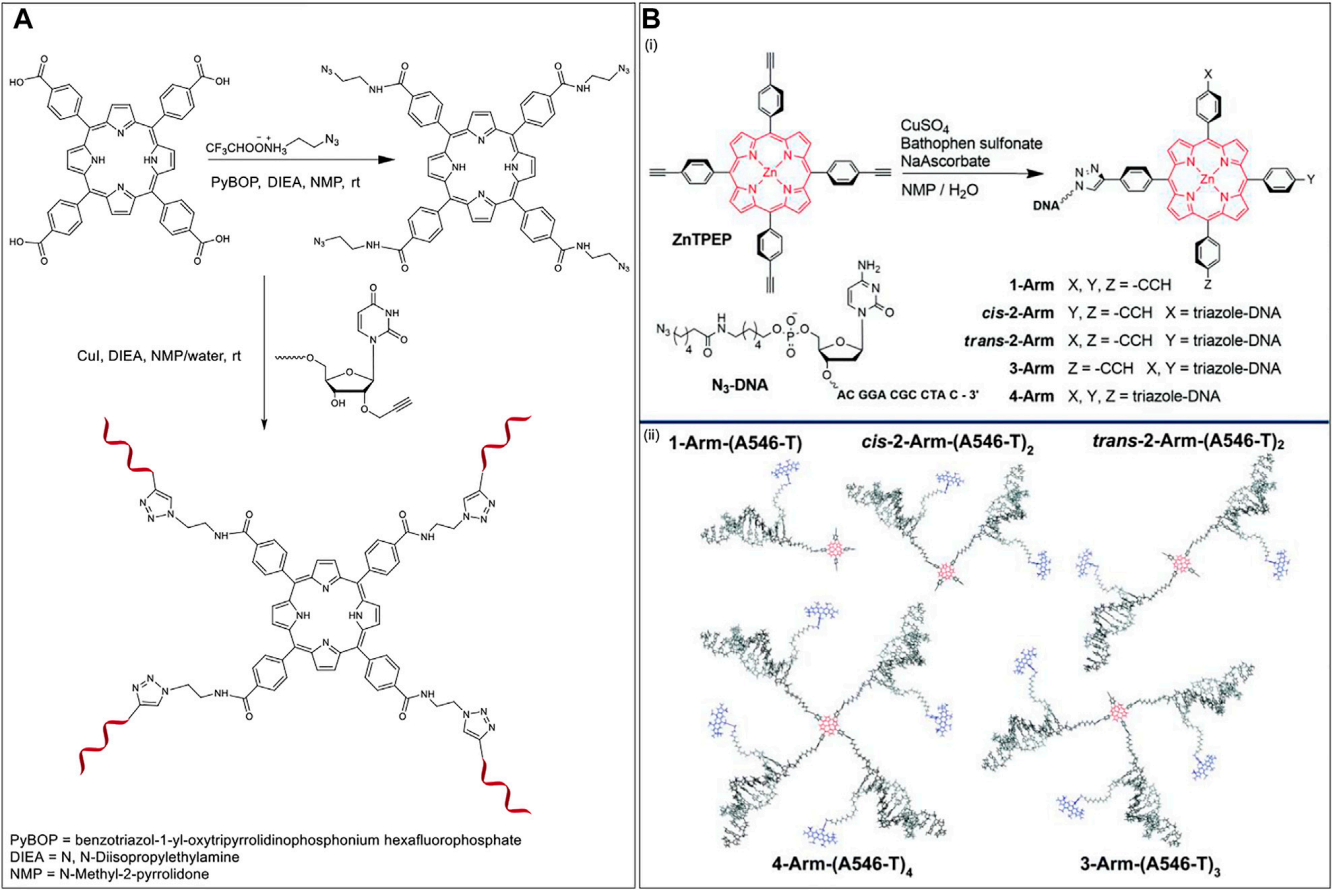
**(A)** Synthesis of multibranched Porphyrin-ON scaffold (Clavé et al., 2014). **(B)** (i) Scheme for the synthesis of multiarm porphyrin-DNA hybrid *via* CuAAC (ii) Hybridization of the porphyrin-DNA hybrids with A546 bearing complementary strands offers various FRET-compatible structures. Reprinted with permission from (Anderson et al., 2018).

In 2014, Campidelli et al. functionalized *meso*-tetra(4-carboxyphenyl)porphyrin with 2-azidoethanamine trifluoroacetic acid salt to obtain the tetra azido derivative of the porphyrin dye (Clavé et al., 2014). The group then synthesized an ON (5′ GGA-GCT-GCA-GTT-CAU-propargyl 3′) to undergo CuAAC with the porphyrin dye to finally achieve a multi-branched porphyrin-DNA scaffold (Figure 6A). The resulting porphyrin-DNA conjugate was sequentially hybridized with a complementary 14-mer DNA strand containing a thiol group at the 3′ end and then incubated with gold nanoparticles (AuNPs). TEM analysis showed the hybrid nanostructures mostly possessing three gold nanoparticles instead of four most likely due to steric hindrances.

In 2018, Wang et al. used a porphyrin-DNA scaffold to develop energy transfer assemblies by tethering different numbers of ON sequences on to Zn-tetra-(phenylethynyl)porphyrin (ZnTPEP) and employing complementary sequences to the ON arms to dial in AlexaFluor 546 (A546) dyes (Figure 6B, top) (Anderson et al., 2018). An azido-modified DNA strand was reacted with the tetra-alkyne modified Zn-porphyrin *via* CuAAC resulting in five different porphyrin–DNA architectures (1-Arm, *cis*-2-Arm, *trans*-2-Arm, 3-Arm, and 4-Arm). Hybridization of the DNA arms with A546 modified complementary sequences led to the formation of the corresponding five different FRET assemblies (Figure 6B, bottom). The donor (A546) and acceptor (ZnTPEP) were shown to display effective FRET, demonstrating the utility of porphyrin-DNA hybrid constructs in light-harvesting applications. The experimental energy transfer efficiencies in the assemblies, 1-Arm, *cis*-2-Arm, *trans*-2-Arm, 3-Arm, and 4-Arm were found to be 75, 72, 85, 87, and 87%, respectively. While the energy transfer might depend on the actual orientation and microenvironment of the fluorophores in the assemblies, it was found that, in general, the energy transfer efficiency increased with an increase in the number of donor dyes in the FRET architecture.

## Porphyrin-Peptide Conjugates

The conjugation of porphyrin derivatives to peptides has found numerous biomedical applications including in PDT, PACT, photochemical internalization (PCI), and PTT. One major reason for attaching the photoactive porphyrin units to peptides is because these biomolecules can direct binding to targeted cell-surface receptors and can induce into-cell translocation. Additionally, the structure of peptides and their self-assemblies can lead to the defined organization of the porphyrin chromophores for emulating biological light-harvesting systems and for interesting optoelectronic devices. As a result of the various potential applications, methods to conjugate porphyrins to peptides have been extensively investigated. Conjugation between porphyrin derivatives and peptides can be classified into two broad categories: 1) ligation based on native side-chain functional groups (thiol, amine), as well as ligation based on N or C-terminus groups; and 2) tethering *via* bioorthogonal reactions (e.g., Staudinger ligation). Amongst these methods, conjugation based on amino groups is the most common, since natural peptides have N-termini and also some amino acids bear nucleophilic amine side chains (e.g., lysine). However, bioorthogonal chemistry provides efficient ligation to unprotected multifunctional peptides and is an area of rapid development (Giuntini et al., 2011). Here, we commence by discussing these bioorthogonal reactions.

### Bioorthogonal Reactions

While conjugation of porphyrin to ONs *via* bioorthogonal reactions is important (*Hydrazide Reaction* and *Copper Catalyzed Alkyne-Azide Cycloaddition and Diels-Alder Cycloaddition* sections), it is even more necessary for peptide-porphyrin conjugation since peptides have many more reactive functional groups. Conjugation of porphyrin derivatives to peptides largely falls under four reaction types. These include Staudinger ligation, CuAAC, Strain promoted alkyne-azide cycloaddition (SPAAC), and ring opening cross metathesis.

#### Staudinger Ligation

**FIGURE 7:**
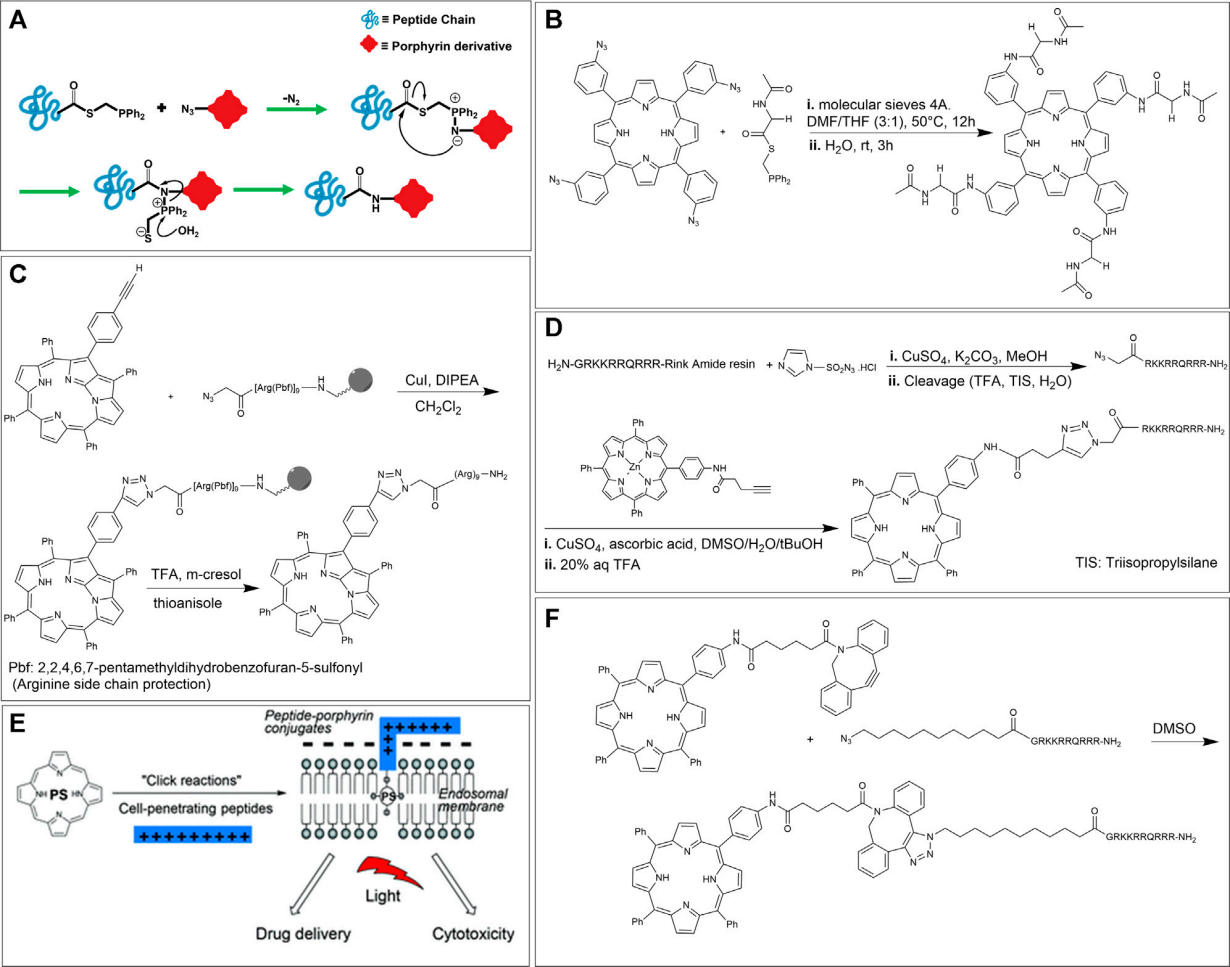
**(A)** Traceless Staudinger ligation mechanism. **(B)** Scheme for conjugation of 5,10,15,20-tetrakis (3-azidophenyl) porphyrin with a phosphine tethered amino acid through Staudinger ligation (Umezawa et al., 2010). **(C)** Conjugation of N-fused tetraphenylporphyrin and nona-arginine peptide by CuAAC on solid support, followed by acid cleavage from the resin (Ikawa et al., 2009). **(D)** CuAAC based bioconjugation of a mono-alkyne functionalized TPP with the corresponding CPP peptide. **(E)** Schematic of PCI using an amphiphilic CPP-targeted photosensitizer. The hydrophobic photosensitizer core localizes inside the lipid bilayer of endosomal/lysosomal vesicles while the cationic peptide resides on the exterior. Reprinted with permission from (Dondi et al., 2016). **(F)** Scheme for conjugation of strained alkyne functionalized porphyrins to the corresponding CPP derivative *via* SPAAC (Dondi et al., 2016).

The conjugation of two components using the mild reaction between an azide and a phosphine is named the Staudinger ligation. This reaction is one of the most important reactions in the bioorthogonal chemistry toolkit since it is rapid and highly chemoselective. While this reaction is compatible with biological conditions, the susceptibility of phosphines for oxidation in the presence of air and their poor water solubility have somewhat limited its scope. The seminal step of the reaction is the formation of a phosphorus ylide and is driven by the expulsion of N_2_. The ylide may subsequently be trapped by a neighboring acyl group to afford a stable amide bond and an attached phosphine oxide. Among the reported Staudinger variations, the traceless method (where, in addition to the formation of an amide bond there is loss of the phosphorus-containing unit) introduced by Raines et al. is particularly amenable for the conjugation of peptides with other entities (Figure 7A) (Nilsson et al., 2001; Bednarek et al., 2020).

In 2010, Umezawa et al. used the traceless Staudinger ligation method for the conjugation of four peptide units onto a porphyrin core. In this paper, the researchers synthesized 5,10,15,20-tetrakis (3-azidophenyl) porphyrin by condensation of 3-azidobenzaldehyde with equimolar pyrrole (Umezawa et al., 2010). Also, the phosphine-containing amino acid partner was prepared according to the literature using a suitable phosphinothiol reagent (Soellner et al., 2002). Last, the ligation of the tetra azido porphyrin to the peptides was carried out in an anhydrous solvent to prevent early-stage hydrolysis. An example of one such reaction is shown in Figure 7B. Here, two equivalents of the amino acid derivative (for each azide group on the porphyrin) were introduced to give the tetra-substituted amino acid-porphyrin conjugate (Umezawa et al., 2010).

#### Copper Catalyzed Alkyne-Azide Cycloaddition

The key features of CuAAC include selectivity (azides are essentially absent in natural biological systems) and applicability (i.e., this reaction works in a broad range of pH and temperature and is significantly faster than the Staudinger ligation). Hence, CuAAC has become an ideal option when considering the ligation of porphyrin photosensitizers to peptides. Indeed, researchers have used CuAAC facilitated porphyrin-peptidic chimeras for applications like PDT (Ke et al., 2012; Sokolova et al., 2013; Acherar et al., 2015; Bryden et al., 2015; Ladomenou et al., 2016; Gazzali et al., 2017), PACT (Feese et al., 2019), and tumor imaging or targeting (Bakleh et al., 2009).

Ikawa et al., in 2009 synthesized a derivative of N-fused porphyrin (NFP) and conjugated this porphyrin with a nona-arginine (R9) peptide tail by “click” chemistry for the first time. As a result, N-fused tetraphenylporphyrin-R9 conjugate was water-soluble and possessed a two-step protonation behavior. The researchers selected NFP as the photosensitizer owing to its absorption of near-infrared (NIR) light over 1,000 nm. This NIR window has been identified as a wavelength suitable in the biomedical field for diagnostic and therapeutic applications due to less interference by tissues, blood, and bio (macro)molecules. Further, R9 is a member of a class of peptides with cell-penetrating ability. The synthesis of N-fused tetraphenylporphyrin-R9 conjugate was carried out by reacting mono-alkyne functionalized N-fused tetraphenylporphyrin with azido modified R9 attached on a Rink Amide resin. Cleavage from the resin was achieved by using trifluoroacetic acid (TFA) (Figure 7C) (Ikawa et al., 2009).

Another example of using CuAAC to generate porphyrin-peptide conjugates was reported by the Eggleston group, in 2016. These researchers used TPP as a template to prepare a mono-alkyne functionalized TPP for light triggered drug delivery and PDT applications. Before conducting the conjugation, zinc metal was utilized to prepare a zinc-porphyrin complex to avoid sequestration of the copper catalyst by the free-base macrocycle. The peptide conjugation partner was synthesized *via* Fmoc based peptide synthesis on Rink Amide resin, using benzotriazol-1-yloxytripyrrolidinophosphoniumhexafluorophos phate (PyBOP) activation. The original peptide was chosen based on the cell-penetrating peptide (CPP) sequence derived from the transcriptional activator (Tat) protein from the human immunodeficiency virus 1 (HIV-1). This decapeptide, known as HIV-1 Tat (48–57), has the sequence GRKKRRQRRR. CPPs are a class of “carrier” systems consisting of 8–30 amino acid residues that can translocate across biological membranes and transport different molecular cargos. In this work, the clickable peptide was synthesized *via* direct diazo transfer on the resin-bound Tat sequence using imidazole-1-sulfonyl azide hydrochloride, followed by cleavage from the resin (Figure 7D) (Goddard-Borger and Stick, 2007). For the CuAAC reaction, the azide functionalized peptide was reacted with an excess amount of mono-alkyne functionalized TPP in the presence of CuSO_4_ and sodium ascorbate in a DMSO/H_2_O/tBuOH mixture, and the requisite N-linked conjugate was successfully obtained after removal of the zinc metal (Dondi et al., 2016).

As an application of this work, in addition to classical PDT, the authors also focused on a novel mechanism of drug activation, namely, PCI. PCI is a photo-induced technique that enables drugs to escape the endosomal and lysosomal pathways and reach the targeted intracellular sites to maximize drug efficacy. In particular, many drugs are uptaken by endocytic mechanisms and are trapped in endo/lysosomes rendering them less effective. In PCI, a photosensitizer can localize into the endo/lysosomal membrane and rupture the membrane *via* singlet oxygen production, as a result of a sub-lethal light dosage. This rupturing event does not directly kill the cells but rather enables entrapped co-drugs to escape and reach their desired target. A critical requirement for such lysosomotropic agents is that they should be amphiphilic. As shown in Figure 7E, the CPP-TPP conjugate has an amphiphilic character as it includes a hydrophobic photosensitizer core (that can embed within the lipid bilayer of endosomal membranes) and a hydrophilic peptide arm that can associate with the exterior of the lipid layer. To confirm uptake and endolysosomal localization of their CPP-TPP conjugate, the authors used confocal microscopy on MC28 rat fibrosarcoma cells and the results showed the predicted sub-cellular localization (Dondi et al., 2016).

#### Strain-Promoted Alkyne-Azide Cycloaddition

While CuAAC reactions are efficient at labeling biomolecules, the conjugation of porphyrins to peptides using this method has some limitations. First, these reactions need to be preceded by a metalation step (typically *via* the pre-formation of the Zn complex). Also, copper catalysts may be toxic to bacterial and mammalian cells, limiting applications of this reaction where cell viability is essential. On the other hand, the conjugation of alkynes with azides can also be catalyzed utilizing ring strain; specifically, strained cyclooctyne partners react with terminal azides without using copper (I) catalysis. However, it should be noted that the latter reactions have slower reaction rate constants compared to CuAAC. (Agard et al., 2004; Lang and Chin, 2014).

The Eggleston group has also investigated the SPAAC strategy for preparing CPP-TPP conjugates. Specifically, the researchers used dibenzocyclooctyne (DBCO) to synthesize mono-DBCO functionalized TPP. The complementary conjugation component, N-terminus azide functionalized CPP, was prepared using a similar strategy developed by this group and discussed above (Figure 7D). In the last step, the reaction of two equivalents of mono-DBCO functionalized TPP with N-terminal azido Tat peptide proceeded in DMSO to give the desired CPP-TPP conjugate (Figure 7F) (Dondi et al., 2016). In addition to demonstrating the expected sub-cellular localization, this CPP-TPP conjugate also showed the highest phototoxicity when compared to other conjugation strategies (e.g., CuAAC, thiol-maleimide, etc.), due to the extended triazole-based linker between hydrophobic porphyrin and the polycationic hydrophilic peptide, which reportedly is highly beneficial. In particular, phototoxicity was examined in monolayer cell culture of MC28 human breast cancer cells and the porphyrin dose required to induce 50% toxicity (LD_50_) after 5 min illumination of blue light was ∼40 nM (compared to μM range when conducted in the dark). These exciting results make this conjugate a potential candidate for PDT applications. Last, to investigate the PCI effect, a protein toxin, saporin (which is a 30 kDa ribosome inactivating protein) was used as the co-drug. These experiments showed that the cell viability was significantly reduced (∼3-fold reduction) when the saporin was also introduced as compared to when the CPP-TPP conjugate alone was illuminated. Importantly, the viability reduction using saporin alone was small (∼10%) (Dondi et al., 2016).

In further work, the same group conjugated TPP derivatives to the C-terminal region of bombesin (BBN [7–14]) using different conjugation strategies including SPAAC, because this polypeptide can target the gastrin-releasing peptide receptor (GRPR), a protein that is overexpressed in several tumors, especially in human prostate cancer (PC-3) cells. First, mono-amino functionalized TPP was reacted with DBCO acid to prepare mono-DBCO functionalized TPP. The polypeptide, on the other hand, was assembled by Fmoc solid phase peptide synthesis on Rink Amide resin. Acylation of the N-terminal amino acid was performed on resin-bound BBN [7–14] using 11 azido undecanoic acid to afford the required azido functionalized polypeptide. Finally, the two components were conjugated *via* SPAAC to prepare TPP-BBN [7–14]. The cell-uptake studies (on PC-3 and HeLa cells) of this conjugate were attempted by confocal microscopy. However, the results showed no uptake in either cell line. Importantly, agglomeration in the cell media was observed (even after several washes with Hank’s Balanced Salt Solution). Hence, a likely cause for the poor uptake many be due to low solubility of the compound (*inter alia*, as a result of the long aliphatic linker). (Ciaffaglione et al., 2021).

#### Olefin Metathesis

**FIGURE 8:**
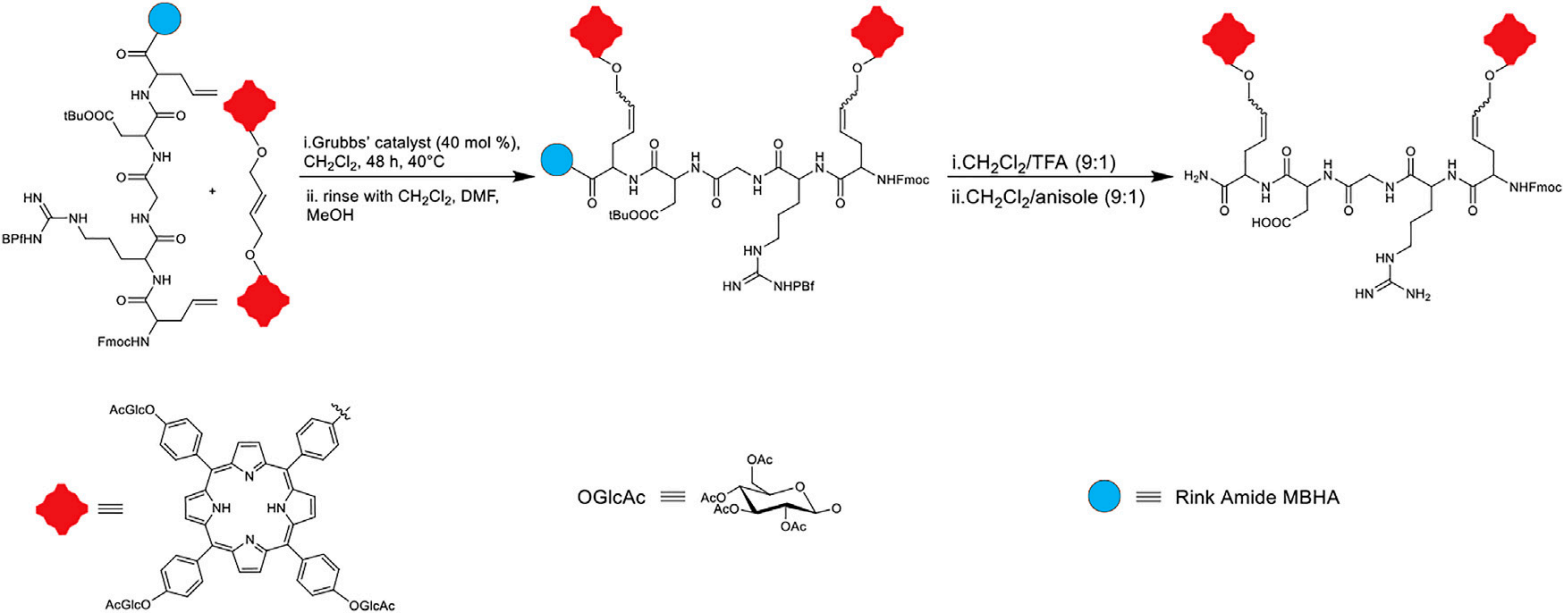
Schematic representation of dimeric porphyrin and peptidic moiety conjugation through olefin cross-metathesis (Sol et al., 2008).

One powerful tool for carbon-carbon double bond formation that is widely used in synthetic chemistry is the metal-catalyzed olefin metathesis. Overall, this reaction involves the scissoring of double bonds of olefins and the distribution of the fragments to produce new carbon-carbon double bonds. The reaction is catalyzed by ruthenium olefin metathesis catalysts such as the well-known Grubbs’ catalysts (Delaude and Noels, 2005; Kirshenbaum and Arora, 2008). Owing to the good activity and commercial availability of Grubb’s catalysts, olefin metathesis has been explored for the labeling of proteins and peptides. In terms of conjugating peptides to porphyrins, Sol and coworkers demonstrated, in 2008, that a glucosylated porphyrin dimer could be conjugated to a pseudo-pentapeptide, containing an arginyl-glycyl-aspartyl (RGD) sequence. In this work, the dimeric triglucosylated porphyrin derivative was synthesized according to previous reports, and the peptide unit was prepared using Fmoc-based solid-phase peptide synthesis. Aiming to insert two porphyrin units on the RGD sequence, the dimeric porphyrin was conjugated to the allylGlycyl-RGD-allylGlycine linear peptide on Rink Amide MBHA resin using the cross-metathesis reaction (Figure 8). Various reaction conditions were tested, including modulating the ratio of reactants, reaction time, temperature, and the use of Grubbs’ I and II catalysts. The best results were obtained when 4 equivalents of the porphyrin unit versus the peptide unit was utilized in the presence of Grubbs’ II catalyst. Upon reaction completion, the product was cleaved and deprotected from the resin (Sol et al., 2008).

The impetus for developing the dimeric porphyrin attached to the RGD peptide was for the potential application in PDT. Here, the authors used the dimeric porphyrin system to increase the amount of photosensitizer that can be activated by light. The two allyl glycine arms were included to provide conformational flexibility to attach porphyrins through olefin metathesis Moreover, the RGD sequence was included since it is established to bind to α_υ_β_3_ integrin (an extracellular matrix protein that is overexpressed in various cancers). Further, the design included the glucosyl units to engender amphipathic character that could enhance passive uptake of the photosensitizer. The sugar appendages may also modulate active uptake of the photosensitizer due to higher glucose uptake by cancer cells. (Chaleix et al., 2004; Chen et al., 2004; Sol et al., 2008).

The efficiency of ^1^O_2_ production was first evaluated by a trapping reaction using ergosterol acetate. In this technique, the ^1^O_2_ production efficacy of the dimeric porphyrin compound was compared to well-known ^1^O_2_ producers, such as hematoporphyrin (HP). The results showed that this conjugate has the same efficacy as HP which means that it could be a promising candidate for PDT applications (Böcking et al., 2000). In addition, the anticancer activity of this dimeric porphyrin conjugate against K562 cells was also evaluated and the photocytotoxicity results showed that dead cell counts in the presence of the dimer were lower than those with Photofrin (a clinical PDT photosensitizer) (Sol et al., 2008).

### Ligation *via* Cysteine Residue

#### Thiol-Maleimide Ligation

**FIGURE 9:**
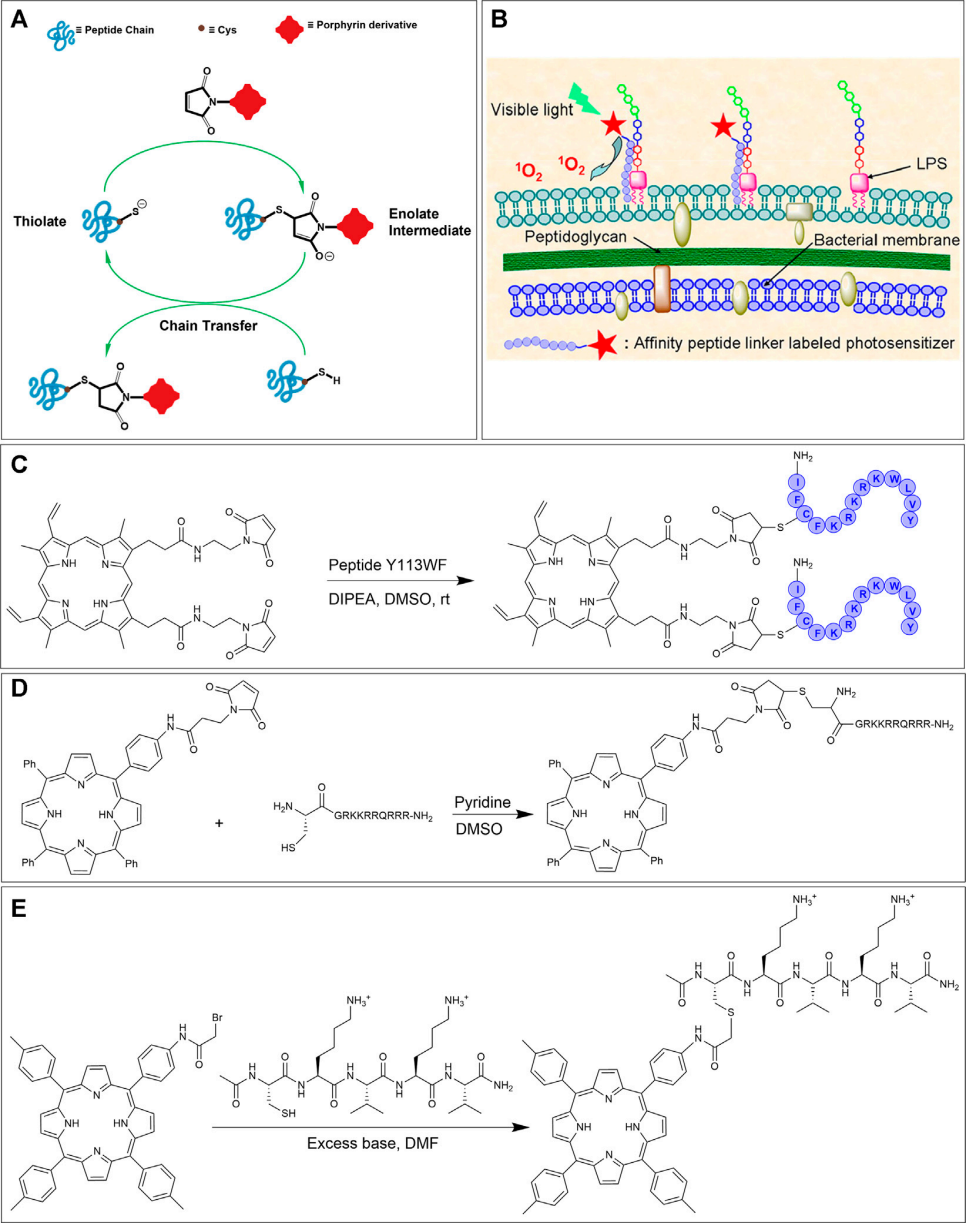
**(A)** Thiol-maleimide ligation mechanism. **(B)** Schematic of the binding of PpIX-Y113WF conjugate to LPS component of Gram-negative bacterial strains. Adapted with permission from (Liu et al., 2012). **(C)** Thiol-maleimide reaction of dimeric protoporphyrin IX and cysteine-containing antimicrobial peptide Y113WF. **(D)** Bioconjugation of maleimide functionalized porphyrin derivative with a thiol tethered CPP (Dondi et al., 2016). **(E)** Synthetic scheme for the preparation of peptide-porphyrin conjugate using the bromoacetamide-cysteine reaction. (Arai et al., 2003).

Maleimide chemistry is an attractive choice for bioconjugation due to the relative ease of accessing functionalized maleimides and their excellent reactivity with thiols. The thiol side-chain containing Cys residues are generally in low abundance within most peptides and proteins, hence making them an ideal choice for fairly selective bioconjugations. In addition, thiol functional groups are significantly superior nucleophiles than amino groups and, in particular, react faster with the double bond of a maleimide under physiological conditions. Taken together, these factors engender thiol-maleimide ligation a salient tool for peptide bioconjugation (Figure 9A) (Wang et al., 2012; Ravasco et al., 2019; Chu et al., 2021).

In 2012, Liu et al. utilized the thiol-maleimide ligation strategy for the attachment of a photosensitizer protoporphyrin (PpIX) to a lipopolysaccharide (LPS) binding antimicrobial peptide Y113WF (YVLWKRKRKFCFI-amide). The goal of this work was to investigate the bioconjugate for PACT activity and intracellular fluorescent imaging (Liu et al., 2012). PACT is a method that utilizes non-toxic (in the dark) photosensitizers to kill microbes by nonspecific oxidative damage by ROS only upon irradiation with light. In fact, energy transfer from the photosensitizer to oxygen can ultimately lead to the production of various reactive oxygen species (H_2_O_2_, OH^●^, O_2_^●−^, ^1^O_2_) that are lethal to microbial pathogens (Bullous et al., 2011; Pereira et al., 2014; Meng et al., 2015; Orosz et al., 2017). Briefly, bis-maleimide functionalized PpIX was reacted with peptide Y113WF in DMSO in presence of DIPEA to achieve dimeric PpIX-Y113WF conjugate. Next, the effectiveness of fluorescent imaging and PACT on Gram-negative bacterial strains based on this conjugation was evaluated (Figures 9B,C). Due to the high binding affinity of the Y113WF peptide sequence to the LPS component, the photosensitizer was able to produce localized ROS. Specifically, these studies demonstrated that the dimeric PpIX-peptide conjugate was ca. 99% lethal to bacteria at 0.5 μM concentration upon light irradiation (30 J/cm^2^). Furthermore, the authors showed that the efficacy of bacterial photoinactivation was concentration-dependent (and could reach 99.9%). As importantly, the dimeric PpIX-Y113WF conjugate also demonstrated the ability to image, in real-time, bacterial strains *via* fluorescence emission. Further photo-inactivation experiments and fluorescence imaging studies illustrated that the dimeric conjugate can selectively target bacterial strains over mammalian cells—an important requisite for PACT (Liu et al., 2012).

Another example of thiol-maleimide ligation was shown by the Eggleston group who conjugated CPPs to TPP. Here, the maleimide functionalized TPP was first synthesized *via* a peptide coupling reaction between mono-amino TPP and 3-maleimidopropionic acid. The complementary thiol-containing peptide was obtained by reaction of Fmoc-L-Cys with resin-bound peptide using HATU activation. In the final step, the N-terminal functionalized cysteine residue on the peptide was reacted with two-fold excess of the maleimide functionalized TPP to offer the CPP conjugate in excellent yield (Figure 9D) (Dondi et al., 2016). After showing, *via* fluorescence microscopy, that the maleimide tethered CPP-TPP conjugate could indeed localize in lysosomes of MC28 rat fibroblasts, the phototoxicity of the conjugate was tested. In particular, illumination of the conjugate in monolayer culture of MC28 cells showed an LD_50_ in the range of 100 nM which was higher than for CPP-TPP conjugates formed by SPAAC (∼40 nM) (*Strain-Promoted Alkyne-Azide Cycloaddition* section) but was much lower than the μM range obtain without illumination. Also, PCI studies showed that the cell viability was significantly reduced (∼three-fold reduction, similar to SPAAC ligation) (Dondi et al., 2016).

#### Thiol-Haloacetamide Ligation

A complementary reaction that can be used to link porphyrinoids to cysteine residues of peptides is the thiol-haloacetamide ligation. In particular, bromo- and iodo-acetamides have been used as electrophilic sites (Chaloin et al., 2001; Arai et al., 2003) with the thiol serving as a nucleophile. Since the thiol on cysteine is more nucleophilic than hydroxyl and amino side-chains on other amino acids, this reaction is selective for cysteine ligation (Giuntini et al., 2011). The principal advantage of thiol-haloacetamide reaction over the thiol-maleimide reaction is the greater stability of the thioether linkage product in the former reaction. The thioether linkage produced from the thiol-maleimide reaction is considered less-stable since it can slowly undergo a retro-Michael reaction to reform the maleimide group which can then react further with other biomolecules. In order to overcome this problem, the thiosuccinimide ring product can be hydrolyzed after conjugation. On the other hand, the thiol-haloacetamide reaction has a slower reaction rate and also requires a higher pH to proceed. This makes the thiol-haloacetamide reaction less chemoselective, because at pH > 7.5 free primary amino groups can compete with thiols to react with the haloacetamide (Schelté et al., 2000; Szijj et al., 2018).

In 2003, Arai et al. tethered a porphyrin to a pentapeptide to mimic natural multiple porphyrin clusters. The pentapeptide sequence (CKVKV) includes alternating hydrophilic and hydrophobic residues and forms a random coil structure in aqueous trifluoroethanol. However, upon conjugation with the porphyrin at the N-terminal cysteine, the pentapeptide assembles into a β-sheet structure due to porphyrin-based pi-stacking. This tendency to form a β-sheet structure is interesting due to its resemblance to natural porphyrin self-assemblies (Chaloin et al., 2001; Arai et al., 2003). The preparation of the bromoacetamide linked porphyrin partner involved first the synthesis of mono-nitro porphyrin using a modified Lindsey method followed by reduction (*via* SnCl_2_) to afford aniline containing porphyrin. Condensation of this porphyrin with bromoacetic acid along with EDC•HCl provided the required porphyrin conjugation agent. The bromo acetamido-linked porphyrin was next reacted with the Cys-containing pentapeptide to afford the porphyrin-peptide conjugate, see Figure 9E (Arai et al., 2003).

### Ligations Through Amino-Groups

Conjugation of native amine functional groups on peptides (typically, the ubiquitous N-terminus or the basic side chain on lysine) with various activated carboxylic acid derivatives or isothiocyanates is a very common method for labeling peptides. These reactions result in the formation of stable amides or thioureas, respectively. Further, selective amino group reaction can be achieved by careful protection of other amines on peptides (Giuntini et al., 2011).

#### Thiourea Formation

**FIGURE 10:**
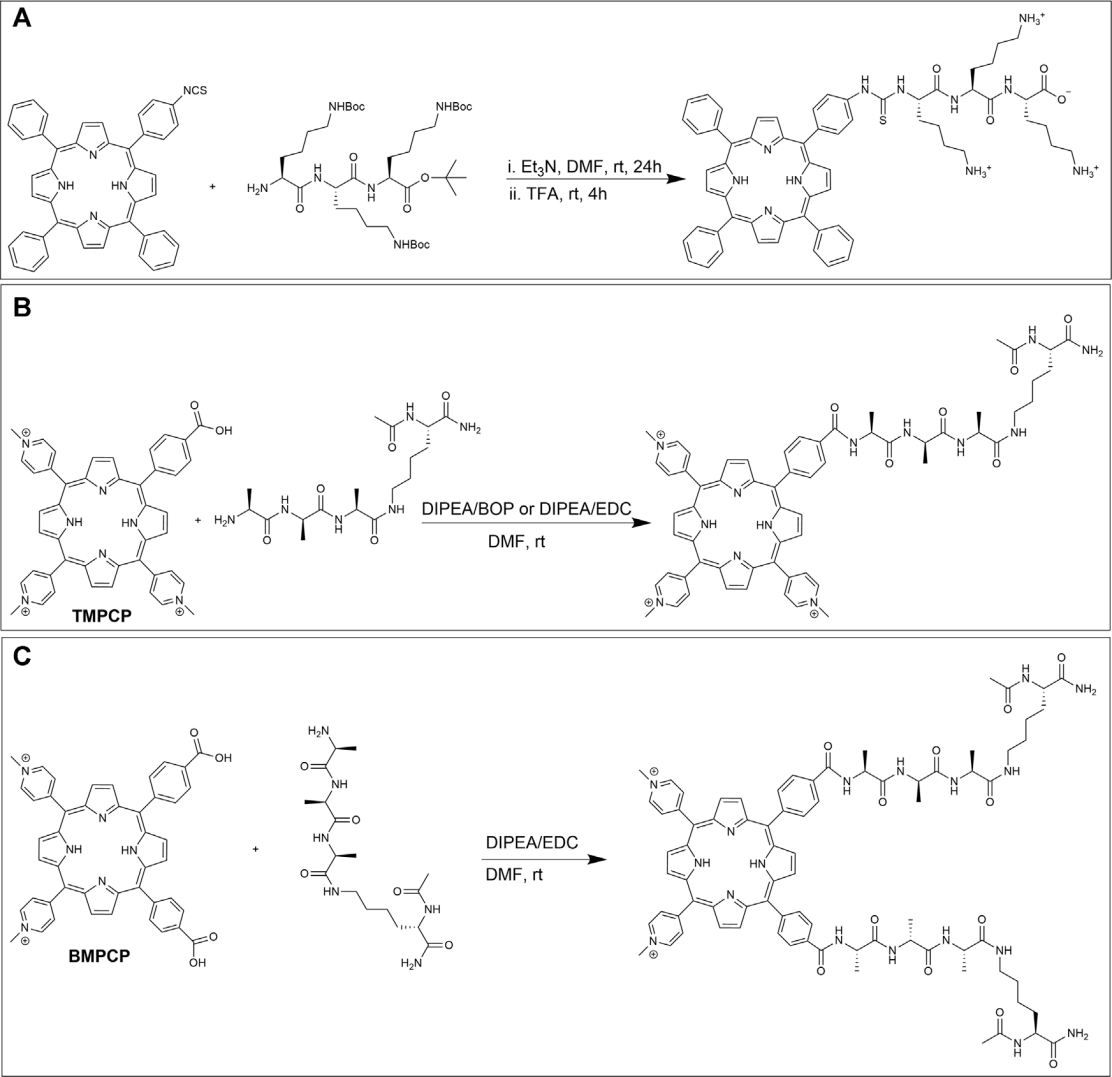
**(A)** Conjugation scheme for a mono-isothiocyanate functionalized porphyrin and Boc protected poly-L-lysine and subsequent deprotection (Sibrian-Vazquez et al., 2005). **(B)** Reaction of TMPCP and one tetrapeptide. **(C)** Reaction of BMPCP and two tetrapeptides by amide linkage formation (Mező et al., 2011).

The reaction of amine groups with activated carboxylic acids can often be slow and lead to the generation of by-products such as N-acyl ureas (Clarke and Boyle, 1999). One method to circumvent these byproducts is to react amines with isothiocyanate derivatives, resulting in thiourea linkages (Smith et al., 2011). Indeed, isothiocyanate (NCS) functionalized porphyrins can be reacted with amines under mild conditions with no by-products formed (Staneloudi et al., 2007). As an example, in 2005, Vazquez et al. conjugated an NCS linked porphyrin to poly-L-lysine to investigate the effect of the number and nature of amino acid conjugates on cellular uptake. Here, the mono NCS-tethered porphyrin was first obtained by exposing mono-amino porphyrin to 1,1′-thiocarbonyldi-(2H)-pyridone. Next, appropriately protected peptides were conjugated to the NCS porphyrin in a mixture of DMF/triethylamine and the requisite product was isolated after deprotection with TFA (Figure 10A). Finally, the dark toxicity (toxicity in absence of light irradiation) and cellular uptake of the porphyrin-peptide conjugate were investigated in human Hep2 cells. The results showed low dark toxicity of this conjugate at a concentration of 10 μM (IC_50_ > 250 μM) (Sibrian-Vazquez et al., 2005).

#### Amide Formation

The reaction between amines on peptides and activated carboxylic acids on porphyrin derivatives have been extensively investigated (Tomé et al., 2004; Gabriel et al., 2007; Giuntini et al., 2011; Shi et al., 2011; Dosselli et al., 2013; Orosz et al., 2013; Li et al., 2015; Jiang et al., 2016; Chen et al., 2018; Liu et al., 2018; Ciaffaglione et al., 2021). For example, in 2011, Mező et al. conjugated one and two tetrapeptides to tri-cationic *meso*-tri (4-N-methylpyridyl)-mono- (4-carboxyphenyl) porphyrin (TMPCP) and bi-cationic *meso*-5,10-bis(4-N-methylpyridyl)-15,20-di- (4-carboxyphenyl)porphyrin (BMPCP) respectively. Their goal was to design porphyrin-peptide conjugates with enhanced cellular uptake and delivery and to facilitate DNA binding. The conjugation partners, TMPCP, BMPCP, and tetrapeptide Ac-Lys (Ala-D-Ala-Ala)-NH_2_ were prepared using standard literature procedures (Habdas and Boduszek, 2005). The amide bond formation was investigated using two different strategies. In the first method, TMPCP and the tetrapeptide were reacted in the presence of DIEA/BOP in DMF (Figure 10B) which resulted in a good yield. While reaction between BMPCP and two tetrapeptides produced many side products. In the second method, EDC was used as the activation reagent in lieu of BOP. Here, the reaction between BMPCP and two tetrapeptides resulted in higher reaction yield (Figure 10C), while no significant improvement was observed for the reaction between TMPCP and a single tetrapeptide. Moreover, DNA binding of porphyrin-peptide conjugates was evaluated by spectroscopic methods due to changes in photophysical properties of bound porphyrins upon interaction with anionic DNA. Here, results from a combination of absorption spectra, fluorescence decay, fluorescence energy transfer, and CD spectroscopy demonstrated that both di- and tri-cationic porphyrins bind to DNA by two different mechanisms including intercalation and external binding. The authors concluded that the tricationic structure and peptide appendages without negative charge are preferable for binding to DNA (Mező et al., 2011).

**FIGURE 11:**
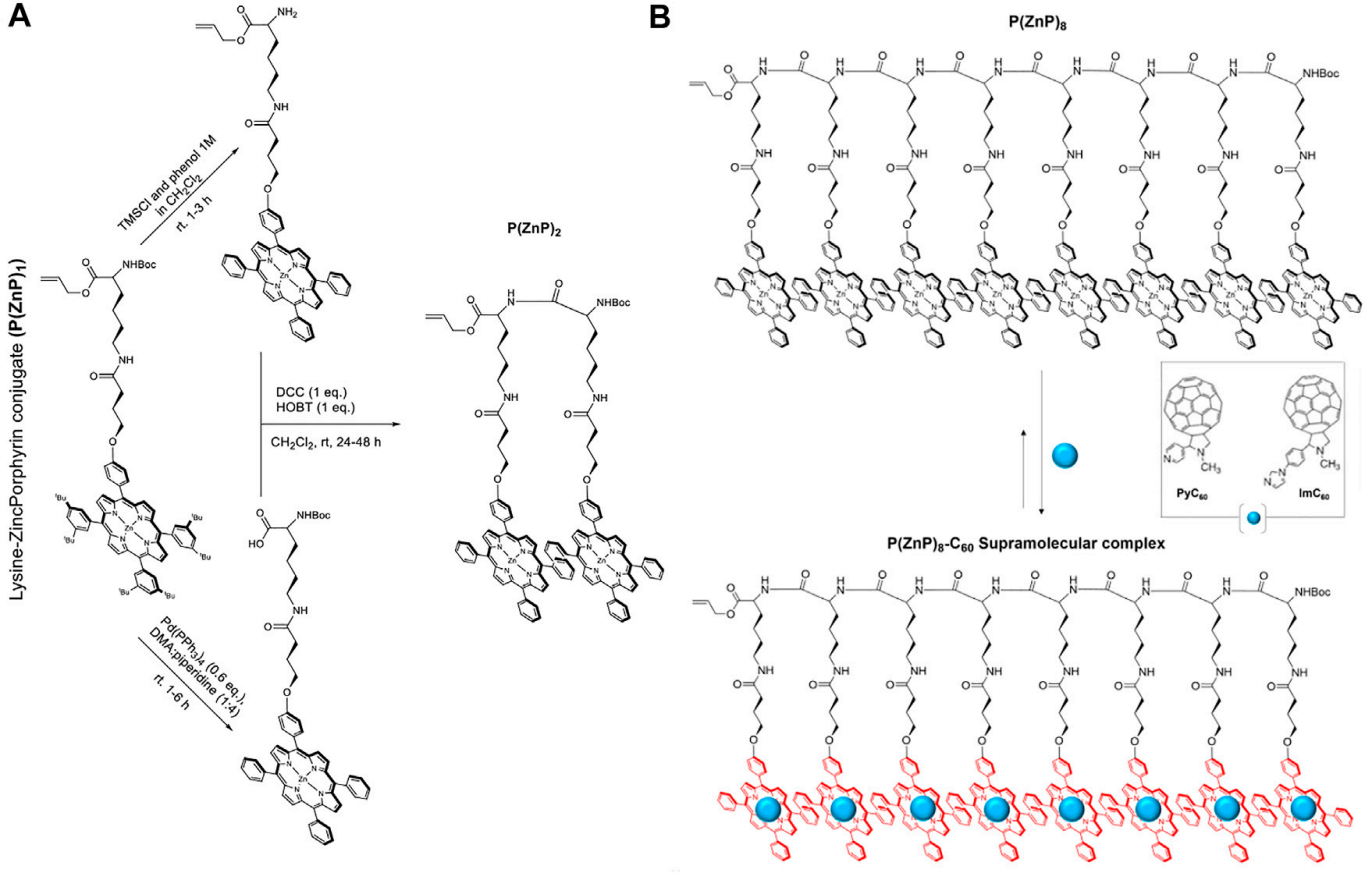
**(A)** General route for oligomerization of (P(ZnP)_1_) (Solladié et al., 2000). **(B)** Illustration of a supramolecular complex composed of a porphyrin–peptide octamer P(ZnP)_8_ and PyC_60_ or ImC_60_ (Solladié et al., 2020).

An active area of research is where porphyrin-biomacromolecule conjugates have applications in artificial light harvesting. Towards this goal, the Solladie group has developed model systems using peptides attached to multiple porphyrins prepared *via* amide coupling (Solladié et al., 2000, 2001; Aubert et al., 2002; Aziat et al., 2008). Their synthetic strategy has focused on *in-situ* carbodiimide-based activation of a carboxylic acid functionality on porphyrins to build a monomer, and subsequent oligomerization *via* further amide couplings. For example, a porphyrin bearing one carboxylic acid arm was reacted with L-lysine (protected with Boc and allyl groups on its N- and C-terminus, respectively) in the presence of N,N′-dicyclohexylcarbodiimide (DCC) and HOBT in DCM. The resulting lysine-porphyrin (**P**(**H**_**2**_ **P)**_**1**_) was metallated with zinc acetate to afford **P(ZnP)**_**1**_. Next, an iterative synthetic strategy was employed to prepare dimers, tetramers, and octamers (Figure 11A). First, one of the coupling partners was prepared by deprotection of the amine group on **P(ZnP)**_**1**_ (with 1 M TMSCl). Next, the other partner was obtained by deprotection of the carboxylic acid end on **P(ZnP)**_**1**_ using tetrakistriphenylphosphine palladium (0). Finally, the amine and carboxylic acid-deprotected versions of **P(ZnP)**_**1**_ were reacted together using DCC and HOBT to afford the porphyrin dimer **P(ZnP)**_**2**_. The syntheses of the tetramer **P(ZnP)**_**4**_ and octamer **P(ZnP)**_**8**_ were carried using similar procedures (Solladié et al., 2000).

Key requirements for artificial light-harvesting systems are efficient light funneling and charge separation processes. Since α-polypeptides can form defined secondary structures (especially α-helices), the multiple porphyrin units could serve as the light capture moieties and the helical peptide conformation could align the porphyrins to promote exciton migration (Solladié et al., 2020). Regarding the charge separation step, a supramolecular strategy was employed wherein the metallated porphyrins could also serve as hosts for fulleropyrrolidines (bearing pyridine, PyC_60_ or imidazole, ImC_60_, sidearms) that are well-known for their electron-accepting capacity (Nair et al., 2014). When the group studied the photophysical capabilities of these complexes (Figure 11B), it was observed that the lifetime of the charge-separated state was enhanced when higher generation oligomers were used, with the **P(ZnP)**_**8**_ **-ImC**_**60**_ ensemble achieving a rather long lifetime of 0.84 ms (Solladié et al., 2020).

**FIGURE 12:**
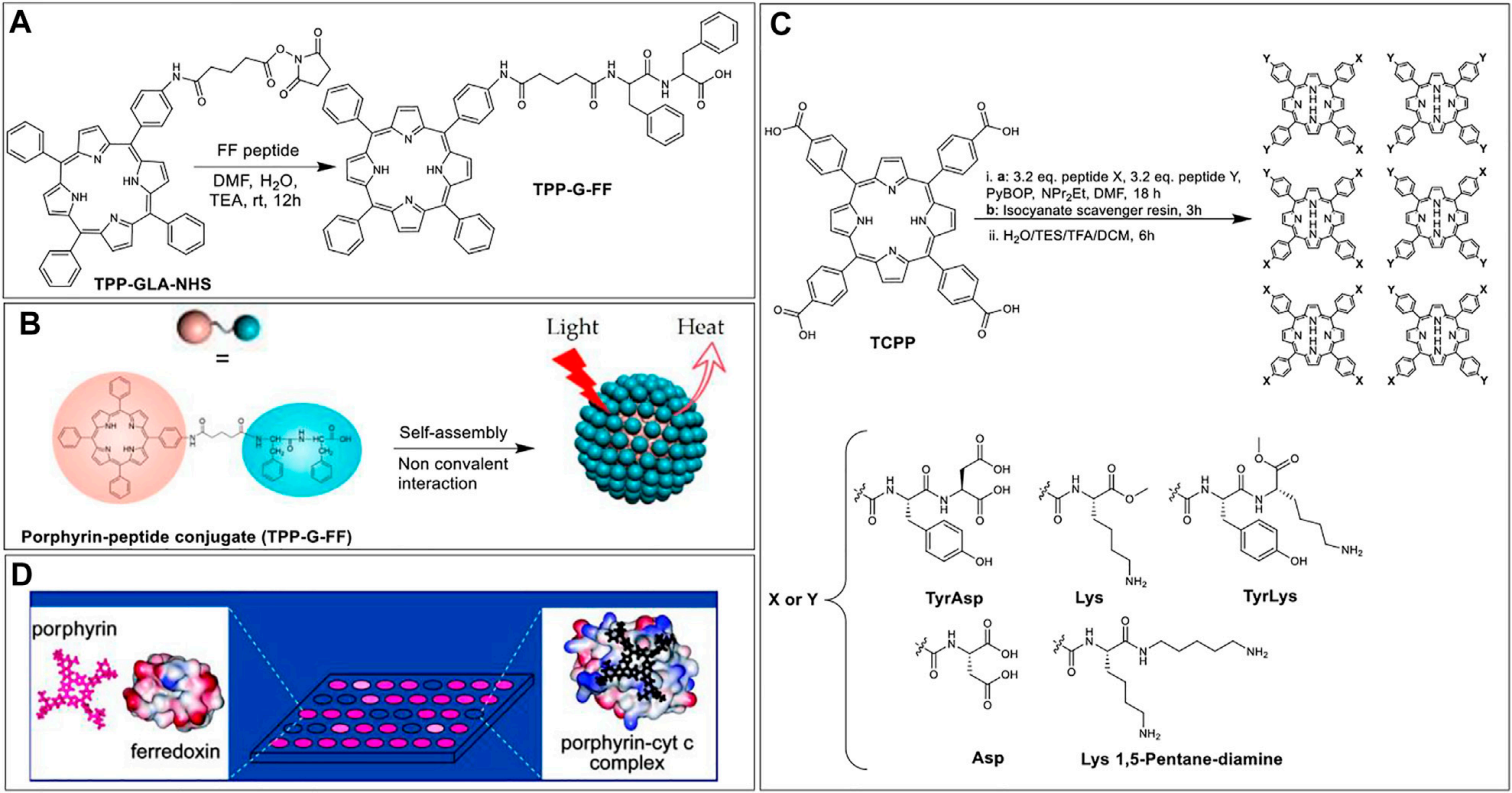
**(A)** Porphyrin NHS ester coupled with FF peptide to afford conjugate TPP-G-FF. **(B)** Schematic illustrating the self-assembly of TPP-G-FF into nanodots. Reprinted by permission from (Zou et al., 2017). **(C)** Scheme of mixed condensation synthesis to form porphyrin based protein-surface binders (Baldini et al., 2004). **(D)** Illustration of a sensing platform where low binding affinity of a porphyrin receptor with ferrodoxin results in negligible fluorescence intensity change (left); while the high binding affinity of the receptor to cyt c results in quenched fluorescence (right). Reprinted by permission from (Baldini et al., 2004).

In addition to *in situ* activation of carboxylic acids, it is often useful to prepare activated, yet stable, carboxylic acid esters that can be stored and used as needed. For example, in 2017, Zou et al. prepared N-hydroxysuccinimidyl esters of glutaric acid tethered porphyrins (TPP-G-NHS) that were then reacted with the L-phenylalanine-L-phenylalanine (FF) dipeptide, under basic conditions to afford peptide-porphyrin conjugate (TPP-G-FF) (Figure 12A). The goal for preparing such conjugates was to self-assemble peptide−porphyrin nanodots (PPP-NDs) for photothermal applications, wherein light energy absorption leads to heat energy release in a controlled manner, resulting in cancer cell death. Notably, this method is different from PDT where the absorbed light energy leads to the generation of ROS (Zou et al., 2017). In this work, the researchers used the FF peptide since it is biodegradable, biocompatible, has functional versatility, and low immunogenicity (Yan et al., 2010). Moreover, FF can co-assemble with different functional molecules through non-covalent interactions to form stable nanomaterials with controllable morphology (Zou et al., 2014, 2016). Nanodot formation was driven by the tendency of hydrophobic porphyrins to form J-aggregates *via* strong π-stacking interactions (Figure 12B). These interactions resulted in the quenching of porphyrin fluorescence thus switching the system towards deactivation *via* heat loss. The PPP-NDs exhibited high stability (even after dilution and irradiation) and afforded a relatively high light-to-heat conversion efficiency (54%). Further, *in vivo* studies on mice models demonstrated that the PPP-NDs accumulate in tumors and are efficient at inhibiting tumor growth.

While most applications of porphyrin-peptide conjugates rely on the photophysical properties of the porphyrins, the Hamilton group elegantly demonstrated that these macrocycles could also be used as scaffolds to project peptides in a defined manner to develop targeted protein surface binders. Indeed, this group has shown that such porphyrin-peptide systems can selectively recognize various protein surfaces (*inter alia via* charge complementarity), including cytochrome c (cyt c), myoglobin, and ferrodoxin (Jain and Hamilton, 2000; Zhou et al., 2006). The porphyrin-peptide protein surface binders are based on tetra-*meso*-carboxylphenylporphyrin (TCPP) as the hydrophobic core. TCPP was conjugated with two different peptides from a pool of five peptides. Specifically, the coupling step involved the reaction of 1 equivalent of TCPP with an excess of the two different peptides in the presence of PyBOP, which yielded six different products for each reaction: X_4_, Y_4_, X_3_Y, XY_3,_ and two geometrical isomers of X_2_Y_2_. These individual products were isolated (and deprotected) for each set of coupling reactions leading to a total of 35 unique porphyrin-peptide conjugates decorated with charges varying from +8 to −8 and having various hydrophobic appendages (Figure 12C) (Baldini et al., 2004). With this sizable pool of porphyrin-peptide derivatives, a rapid screening strategy was developed (based on the porphyrin fluorescence intensity change) to identify high-binding-affinity porphyrin receptors for the targeted proteins (Baldini et al., 2004; Zhou et al., 2006). Figure 12D shows an illustration representing the sensing platform where strong binders are fluorescence quenched. From these experiments, the authors found that charge complementarity was critical with e.g., ferrodoxin interacting with positively charged porphyrin conjugates such as porphyrin-Lys_2_-Lys 1,5 pentadiamine_2_ whilst cytochrome c shows higher binding to the negatively charged receptors (e.g., porphyrin-Asp_4_).

## Conjugation to Proteins

Similar to shorter peptide sequences, the amine side chain of lysine and the thiol moiety of cysteine are the two attractive functional groups for conjugating porphyrins to native proteins. More recently, various biotechnological tools have been developed to modify native amino acids or introduce unnatural amino acids to access further functional handles (Kordbacheh and Kasko, 2021). While some site-selective conjugations are possible due to advancement in protein engineering techniques, labeling proteins with small-molecule tags/payloads, in general, results in heterogenic distribution of the tag with low regio-selectivity (Agarwal and Bertozzi, 2015; Shadish and DeForest, 2020). Hence, these conjugations often lead to a wide distribution of species with a different number of small molecules per protein molecule. Another issue is that proteins tend to aggregate/precipitate and/or denature under unfavorable changes in temperature, pH, shear stress, ionic strength, and solvent environment (Cromwell et al., 2006; Wang et al., 2010). These issues can be more pronounced when a hydrophobic tag such as a porphyrinoid is attached. As a result of the abovementioned problems, there have been fewer reports of porphyrin-protein conjugates compared to porphyrin-peptide or porphyrin-DNA conjugates. However, a specific subfield that is generating increased attention is the conjugation of porphyrins to antibodies (*vide infra*). Many of the issues with protein-porphyrin conjugations can be mitigated by employing antibodies as the protein portion because antibodies are relatively more stable than many proteins and have a well-defined structure. Further, antibodies are relatively easy to scale up in large quantities and are amenable to enzymatic modifications (Bullous et al., 2011).

**FIGURE 13:**
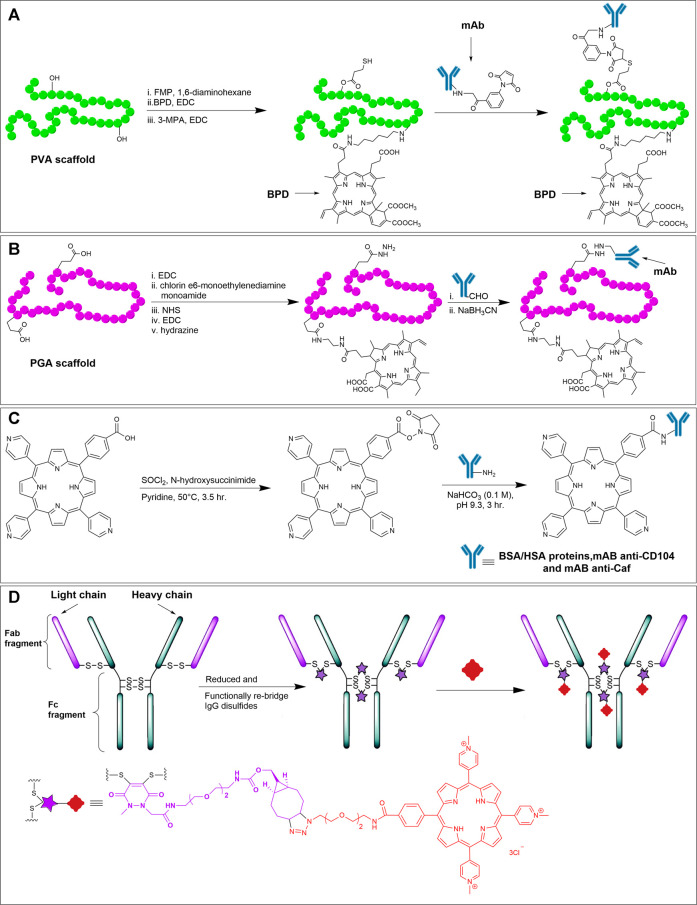
**(A)** Synthetic scheme for mAb-BPD conjugate on a PVA scaffold (Hemming et al., 1993). **(B)** Synthesis of mAb (OC125)-chlorin e6 conjugate on a PGA scaffold (Goff et al., 1994). **(C)** Reaction scheme showing the NHS activated carboxylic acid on *meso*-tri (4-pyridyl)-mono- (4-carboxyphenyl) porphyrin, and subsequent conjugation with amines on albumins (Porphyrin-BSA and Porphyrin-HSA) or mAbs (anti-CD104 and anti-Caf) (Pereira et al., 2014). **(D)** Reduction of the disulfides followed by functional re-bridging with the bifunctional linker generates the IgG with four strained cyclic alkynes, which react with azido TMPyP derivative to offer the antibody-porphyrin conjugate. Adapted with permission from (Maruani et al., 2015).

Antibodies are a special class of Y-shaped glycoproteins that are generated by the immune system to selectively detect and neutralize foreign bodies (Chiu et al., 2019). Antibodies are composed of light and heavy polypeptide chains kept intact in shape by various supramolecular interactions, along with the covalent interchain disulfide bonds. Out of various isotypes and subclasses, Immunoglobulin G1 (IgG1) is the most abundant and important class of antibodies. In IgG1, there are two identical light chains and two identical heavy chains (see Figure 13D). The light and heavy chains are connected *via* one disulfide bond, whereas the two heavy chains are connected *via* two disulfide bonds. The upper part of the antibody (where light chains interact with the heavy chains) possesses antigen-binding fragments (Fab), in which the two antigen-binding sites are located at the tip of the variable domain (F_v_). The stem of the Y-shaped structure, which is the constant domain of the heavy chains is called the crystallizable fragment region (Fc). The Fc region of the antibody also bears glycans on each heavy chain.

Importantly, carefully developed antibodies can have sub-nanomolar to picomolar binding affinity towards target antigens. Because there are antibodies identified to selectively target cell-surface receptors overexpressed in cancer cells, antibodies by themselves have been used for cancer therapy (Zahavi and Weiner, 2020). However, in the past few decades, much interest has been shifted towards developing Antibody-Drug Conjugates (ADCs) for targeted therapy (Ducry and Stump, 2010; Agarwal and Bertozzi, 2015; Gordon et al., 2015; Zhao et al., 2020). Conjugating cytotoxic payloads to antibodies *via* appropriate linkers greatly enhances the utility of such ADCs. Provided that the linker connecting the antibody and drug is stable in serum and undergoes input-responsive cleavage inside the cytoplasm (e.g., low pH inside lysosomes, free thiols to cleave disulfides, protease enzymes to cleave peptide bonds, etc.), ADCs are potent and target-specific because the antibody acts as a vehicle to transport the toxic payload to the target site, where the linker dissociates releasing the free drug moiety (Nolting, 2013). Even if the linker is non-cleavable, the drug can be released by enzymatic degradation of the whole antibody.

Since antibodies have high selectivity and affinity to their targets, hybrid porphyrin-antibody conjugates can be used as ADCs for targeted therapeutic applications, where the porphyrinoid acts as a photosensitizer at the local site targeted by the antibody. Such antibody-photosensitizer conjugates are also referred to as photoimmunoconjugates. Although a handful of ADCs (12, at the time of this manuscript prepration) are approved by FDA (and a few dozen more in clinical trials) to treat various cancers, none of the photoimmunoconjugates are yet approved, to our knowledge (Zhao et al., 2020). However, a plethora of reports discussing attempts, results, and potential of photoimmunoconjugates have been published recently. Here, we discuss a few representative examples of antibody-porohyrinoid conjugates. There are also some excellent reviews that detail the historical development and challenges of photoimmunconjugates (van Dongen et al., 2004; Bullous et al., 2011; Pereira et al., 2015; Fernandes et al., 2019; Sandland and Boyle, 2019).

Antibodies have been functionalized with porphyrinoids *via* common conjugation strategies as discussed in earlier sections. In terms of lysine conjugation, the amino group of a lysine residue of the antibody reacts with porphyrin functionalized with carboxylic acid, NHS ester, isothiocyanates, or other activated esters. However, there are 80–90 accessible lysine residues in a typical IgG1. (Gordon et al., 2015). Thus, lysine conjugation products are exceedingly difficult to control in terms of porphyrin loading ratio and site-specificity. There is also a risk of the antigen-binding region being hindered after the conjugation. In cysteine conjugation, the interchain disulfide bridges in antibodies are first reduced with reducing agents like tris(2-carboxyethyl)phosphine or dithiothreitol. The generated free thiols can then reacted with maleimide, α-halo ester, acryloyl, or pyridyl disulfide functionalized porphyrins (Bullous et al., 2011). Since there are 4 interchain disulfide bridges in IgG1, the tethered products will have porphyrin-to-antibody ratios up to 8 depending on the exact reduction conditions and molar ratios used. In addition to tethering *via* lysine and cysteine residues, the glycans in the Fc domain of the IgG can also be oxidized to generate aldehydes for subsequent bioorthogonal reductive amination with amino-modified porphyrinoids (Xiao-Ming et al., 1992). There are also reports of employing protein-tag technologies to generate site-specific photoimmunoconjugates conjugates (e.g., SNAP-tag) (Hussain et al., 2011). SNAP-tags are 20 kDa proteins that react specifically and rapidly with benzylguanine derivatives and can be expressed as a fusion protein with the antibody intended for labeling.

The development of photoimmunoconjugates was pioneered by Levy et al. in the 1980s and 1990s (Mew et al., 1983, 1985; Jiang et al., 1991). In the first endeavor to conjugate a porphyrin to a monoclonal antibody (mAb), Levy et al. tethered anti-M-1 mAb with hematoporphyrin (Mew et al., 1983). Anti-M-1 has specificity for DBA/2J myosarcoma M-1. The conjugation was achieved *via* EDC coupling between the lysine side chain of anti-M-1 with the carboxyl moiety of hematoporphyrin. *In vivo* studies showed that at a much lower dosage (0.268 mg/kg body weight) of the anti-M1-hematoporphyrin conjugate compared to that in the earlier studies (2.5–5.0 mg/kg body weight) involving hematoporphyrin alone was able to significantly reduce tumor growth in mice. The tumor reduction was significant only with the treatment plan that involved the conjugate irradiated with light. In 1985, the Levy group also conjugated hematoporphyrin to CAMAL-1 monoclonal antibody that has specificity for a leukemia-associated antigen (CAMAL). *In vitro* tests showed that the CAMAL-1:hematoporphyrin conjugate was able to efficiently kill CAMAL expressing cells when irradiated with a 620 nm dye laser. A similar conjugation method was followed by Németh et al., in 1991 (Berki and Németh, 2005) and Pass et al., in 1993 (Pogrebniak et al., 1993) to treat human gastric cancer and lung cancer, respectively, in athymic mice. Further, Németh et al., in 1998 again used the same carbodiimide conjugation strategy to generate a-Thy-1-hematoporphyrin antibody targeting Thy-l antigen in T lymphocytes to show the selective killing of T lymphocytes by the phototoxic conjugate upon irradiation (Berki and Németh, 1998). Other groups have also used similar carboxyl modified commercially available photosensitizers, and have performed amide coupling with antibodies for targeted tumor cell killing (Roberts et al., 1987; Martsev et al., 1995; Linares et al., 2004; Deonarain et al., 2012; Konopińska et al., 2015). Various polymeric linkers (polyvinyl alcohol, polyglutamic acid, dextran, and polylysine) have also been incorporated to generate photoimmunoconjugates (Bullous et al., 2011). These polymeric linkers help enhance the physicochemical properties of the conjugates but can result in a non-specific and random distribution of the photosensitizers on the antibodies.

Hemming et al., in 1993 conjugated a benzoporphyrin derivative (BPD) to tumor-specific anti-epidermal growth factor receptor (anti-EGFr) antibody (Hemming et al., 1993). First, a polyvinyl alcohol scaffold (PVA) was activated with 2-fluoro-1-methyl pyridinium toluene-4-sulfonate (FMP) and then reacted with 1,6-diaminohexane (Figure 13A). The amino-modified PVA was subjected to EDC mediated coupling with BPD to generate a PVA-BPD construct. Further, the PVA-BPD construct was functionalized with free thiols by reacting with 3-mercaptopropionic acid (3-MPA). The mAb was functionalized to bear a maleimide group by reacting with *m*-maleimido-benzoyl-*N*-hydroxysulfo-succinimide ester. Finally, the thiol-maleimide coupling led to the formation of antibody-porphyrin conjugates. Compared to the control groups (treated with light alone or non-specific antibody-porphyrin conjugates), 80% of animals (Syrian golden hamsters) showed marked tumor necrosis and were disease-free for 1 month.

As discussed in *Hydrazide Reaction* section, hydrazide-aldehyde reaction to generate a hydrazone bond is a viable bioorthogonal method and has been used to functionalize antibodies because the Fc region of IgGs are naturally glycosylated and thus can be oxidized to afford the aldehyde functionality. Hasan et al., in 1994 conjugated a chlorin derivative to a murine mAb, OC125, that has specificity for the CA125 antigen (overexpressed in non-mucinous ovarian tumors) (Goff et al., 1994). First, a polyglutamic acid (PGA) scaffold was loaded with a chlorin derivative (chlorin e_6_ monoethylenediamine) *via* carbodiimide coupling followed by the hydrazide modification of the carboxylic acid of PGA (Figure 13B). The oligosaccharide in the mAb was oxidized to offer an aldehyde moiety. Finally, the chlorin-PGA-hydrazide construct was reacted with aldehyde functionalized mAb to form a hydrazone linkage, which was further reduced with sodium cyanoborohydride to yield a stable photoimmunoconjugate. Subsequent photo-irradiation studies suggested that multiple low-dose PDT was able to improve efficacy and minimize the toxicity of the conjugate.

The Boyle group has been at the forefront of antibody-porphyrin conjugate development in recent years. For instance, in 2005, Boyle et al. conjugated 5-(4-isothiocyanatophenyl)-10,15,20-tri-(3,5-dihydroxyphenyl)porphyrin and 5-(4-isothiocyantophenyl)-10,15,20-tris-(4-N-methylpyridiniumyl) porphyrin to murine mAbs 35A7 and FSP 77 *via* amine-isothiocyanate coupling (Hudson et al., 2005). mAb 35A7 recognizes carcinoembryonic antigen (CEA), which is overexpressed in colon adenocarcenomas. Similarly, mAb FSP 77 recognizes the extracellular domain of the erb-B2 receptor, which is overexpressed in ovarian and breast cancer. An *in vivo* biodistribution study showed that the tumor/normal tissue ratio for the conjugates was 33.5 and 13.8 for 35A7-porphyrin and FSP 77 conjugates, respectively. In 2010, the same group conjugated isothiocyanate functionalized cationic porphyrins to tumor-specific monoclonal antibodies, anti-CD104, anti-CD146, and anti-CD326 (Smith et al., 2011). With PDT, these conjugates exhibited the same potency as commercial PDT agent, Photofrin, in reducing human LoVo tumor in mice but at a much lower dosage (10 nmol/kg vs 8.3 μmol/kg).

In 2014, Pereira et al. reported the conjugation of a novel *meso*-tri (4-pyridyl)-mono-(4-carboxyphenyl) porphyrin with four different proteins using N-hydroxy-succinimide-amine couplings (Pereira et al., 2014). Briefly, the conjugation of NHS ester modified porphyrin with surface accessible amines (usually lysine) of bovine and human serum albumins (BSA and HSA) and mAb anti-CD104 and anti-Caf was conducted in an aqueous, slightly basic pH buffer (Figure 13C). UV-Vis spectroscopy was employed to evaluate the degree of porphyrin labeling (DOL). These studies showed that the highest DOL was obtained for a 30:1 initial molar ratio of porphyrin to protein (leading to ∼1 and ∼2 porphyrins attached to BSA and HSA conjugates, respectively). Employing a higher molar ratio for both conjugates showed a decrease in DOL number due to two reasons. First, an insufficient number of available albumins for conjugation, and second, a decrease in albumin solubility at higher concentrations of porphyrin. For porphyrin-mAb anti-Caf and porphyrin-mAb anti-CD104 conjugates, the highest DOL number were 0.81 and 0.80, respectively (Pereira et al., 2014).

Despite lysine conjugations being commonplace for labeling antibodies, attempts to employ more site-selective cysteine conjugations and bioorthogonal chemistries have also advanced. For instance, Maruani et al., in 2015 synthesized a bifunctional linker bearing dibromo-pyridazinedione and ring-strained cyclic alkyne units (Maruani et al., 2015). The linker was judiciously designed for harnessing the reactivity of thiols (generated after reducing 4 interchain disulfide bonds) and the use of SPAAC chemistry. An FDA-approved mAb, trastuzumab (that recognizes HER2 receptor in breast cancer) was reduced with TCEP to generate 8 free thiols (4 pairs previously forming the disulfide bridges) (Figure 13D). Each pair of free thiols reacted with the dibromo-pyridazinedione unit forming a new bridge with the linker species. Here, the dibromo-pyridazinedione reacts with the two thiols generated by reduction of each disulfide bond (thus re-bridging the disulfide bond) such that the mAb bears strained alkyne functionalities at the interchain disulfide locations. SPAAC reaction between the modified mAb and an azido functionalized water-soluble TMPyP derivative yielded the mAb-porphyrin conjugate with a photosensitizer-to-antibody (PAR) ratio of 4. The conjugate was very efficient in eradicating the HER2+ cell lines when irradiated with 625 nm light.

## Conclusion

Nature has evolved proteins as structural and functional scaffolds to fine-tune the photophysical and photochemical abilities of porphyrinoids leading to key processes that drive life on Earth. Inspired by these elegant pigment-protein systems, but with the goal of generating stable, soluble, and non-aggregating entities, with potential applications in materials and biomedicine, researchers have developed strategies to covalently connect porphyrinoids to biomacromolecules. Here, we have surveyed the tethering reactions employed to join porphyrins with ONs or peptides/proteins. The precise reaction route adopted depends on the nature of the biomolecule. Further, the type, quantity, and accessibility of native or artificially introduced functional groups will dictate the effectiveness of the coupling and the homogeneity of the product.

In general, DNA-porphyrin conjugations typically employ orthogonal functional groups that are chemically introduced onto the ON prior to conjugation since ONs do not possess readily reactive nucleophilic moieties. The reactions utilized range from solid-phase phosphoramidite chemistry to various post-synthesis methods (e,g., amide couplings and cycloaddition reactions). In contrast, peptides have more reactive functionalities (amine, thiol, carboxylic acids) that allow for direct conjugation with compatibly functionalized porphyrinoid partners. However, due to the multitude of similar functional groups present on peptides, these reactions typically require judicious functional group protection. To address site-selectivity issues, bioorthogonal chemistry has been employed (e.g., CuAAC, Staudinger ligations, olefin metathesis) in conjunction with site-specific labeling of reaction partners. Moreover, bioorthogonal reactions are continuously being fine-tuned so as to be faster, non-toxic, and non-interfering with other biological processes and metabolites.

In terms of applications, the porphyrin-ON conjugations have largely been used for programmed nanostructure self-assembly due to the high-fidelity molecular recognition properties that DNA sequences offer. The self-assemblies have enabled the precise positioning of porphyrinoids within DNA duplexes and higher-order structures (quadruplexes, DNA-porphyrin nanoparticles, DNA-lipid complexes, etc.). These systems have been evaluated in terms of fundamental properties (chiro-optical, energy transfer, porphyrin aggregation, helix-stabilization) but also for more translational potential such as anti-microbial materials, site-specific nucleic acid cleavage, and light-harvesting systems). One drawback of conventional ONs is their potential to be degraded by natural nucleases, and thus an emerging avenue involves employing Peptide Nucleic Acids (PNAs; molecules mimicking ONs with a peptide backbone instead of the sugar-phosphate skeleton) as the conjugation partners (Nielsen, 1999; Accetta et al., 2015; Nikoloudakis et al., 2019; Chang et al., 2020). The absence of the negatively charged phosphate group and a relatively flexible backbone help PNAs bind with ONs (for site-specific DNA damage) with increased affinity and specificity.

Peptide-, and especially, antibody-porphyrinoid conjugates have major applications in the biomedical field, especially for targeted treatment involving light-activation of the porphyrinoid (PDT, PACT, PTT). Here, the proteinaceous moiety often provides solubility and minimizes the aggregation of the photosensitizer whilst also enabling site-specific targeting (*via* binding to cell-surface receptors and facilitating internalization). Although much ground has been covered towards the development of clinically viable peptide/antibody-porphyrin conjugates, more work needs to be done in the design and optimization of input-responsive cleavable linkers. Such linkers are likely to enhance the efficacy of the peptide/antibody-porphyrin conjugates by ensuring the efficient release of the photosensitizer payloads at the target site. Further improvement in the performance and therapeutic index of photoimmunoconjugates could be achieved by incorporating site-specific conjugation technologies such as engineered amino acids (e.g., THIOMAB), and peptide tags to mitigate the issues of heterogeneity (Junutula et al., 2008; Agarwal and Bertozzi, 2015). Last, a new direction that is gaining attention is the dual-function systems wherein researchers have designed conjugates bearing radioactive, fluorescent, or photosensitizer molecules for intraoperative radiodetection, fluorescence imaging, and targeted photodynamic therapy. Here, either radio- and fluorescent-labeled cancer cell-targeting antibodies are used for image-guided surgery for complete resection of tumor lesions (Hekman et al., 2017; Derks et al., 2021) or chromophore-tagged antibodies introduce tumor-targeted photocytotoxicity and resolution of residual micrometastases (Spring et al., 2014).

## Glossary

ADCs: antibody-drug conjugates
Anti-EGFr: anti-epidermal growth factor receptor
AsPh3: triphenylarsine
AuNPs: gold nanoparticles
BMPCP: *meso*-5,10-bis(4-N-methylpyridyl)-15,20-di- (4-carboxyphenyl)porphyrin
BOP: benzotriazol-1-yloxytris(dimethylamino)phosphonium hexafluorophosphate
BPD: benzoporphyrin derivative
BSA: bovine serum albumin
CEA: carcinoembryonic antigen
CD: circular dichroism
CPP: cell-penetrating peptide
Cyt c: cytochrome c
CuAAC: copper catalyzed alkyne-azide cycloaddition
DABCO: 1,4-diazabicyclo[2.2.2]octane
DBCO: dibenzocyclooctyne
DCC: N,N′-dicyclohexylcarbodiimide
DCM: dichloromethane
DIPEA: N,N-Diisopropylethylamine
DMAP: 4-Dimethylaminopyridine
DMF: Dimethylformamide
DMT: dimethoxytrityl
DNA: deoxyribonucleic acid
DMSO: Dimethyl sulfoxide
DOL: degree of labeling
EDC: 1-Ethyl-3-(3-dimethylaminopropyl)carbodiimide
FMP: 2-fluoro-1-methyl pyridinium toluene-4-sulfonate
GRPR: gastrin-releasing peptide receptor
HATU: 1-[Bis(dimethylamino)methylene]-1H-1,2,3-triazolo[4,5-b]pyridinium 3-oxide hexafluorophosphate
HIV-1: human immunodeficiency virus 1
HOBT: hydroxybenzotriazole
HP: hematoporphyrin
HSA: human serum albumin
IgG1: immunoglobulin G1
LPS: lipopolysaccharide
mAb: monoclonal antibody
NCS: isothiocyanate
NEt_3_: triethylamine
NFP: N-fused porphyrin
NHS: *N*-hydroxy succinimide
NIR: near-infrared
ONs: oligonucleotides
PACT: photodynamic antimicrobial chemotherapy
PAR: photosensitizer-to-antibody ratio
PCI: photochemical internalization
PC-3: prostate cancer cells
PDT: photodynamic therapy
PGA: poly glutamic acid
PMβCD: per-*O*-methylated β-cyclodextrin
PPP-NDs: peptide-porphyrin nanodots
PTT: photothermal therapy
PVA: polyvinyl alcohol
PyBOP: benzotriazol-1-yloxytripyrrolidinophosphonium hexafluorophosphate
SPAAC: strain-promoted alkyne-azide cycloaddition
TCPP: tetra-*meso*-carboxylphenylporphyrin
TFA: trifluoroacetic acid
THF: Tetrahydrofuran
TIPS: triisopropylsilyl ether
TMPCP: tri-cationic *meso*-tri (4-N-methylpyridyl)-mono- (4-carboxyphenyl) porphyrin
TMPP: *meso*-tetra-(4-*N*-methylpyridyl)porphyrin
TMPyP: *meso*-tetrakis(N-methyl-4-pyridyl)porphine tetrakis(p-toluenesulfonate)
TMSCl: trimethylsilyl chloride
TPP: *meso*-tetrakistetraphenylporphyrin
ZnTPEP: Zn-tetra-(phenylethynyl)porphyrin
